# Comparative study on differential expression analysis methods for single-cell RNA sequencing data with small biological replicates: Based on single-cell transcriptional data of PBMCs from COVID-19 severe patients

**DOI:** 10.1371/journal.pone.0299358

**Published:** 2024-03-27

**Authors:** Jie Xue, Xinfan Zhou, Jing Yang, Adan Niu

**Affiliations:** Department of Statistics, School of Economics, Hangzhou Dianzi University, Hangzhou, China; Ege University, Faculty of Medicine, TURKEY

## Abstract

Single-cell RNA sequencing (scRNA-seq) is a high-throughput experimental technique for studying gene expression at the single-cell level. As a key component of single-cell data analysis, differential expression analysis (DEA) serves as the foundation for all subsequent secondary studies. Despite the fact that biological replicates are of vital importance in DEA process, small biological replication is still common in sequencing experiment now, which may impose problems to current DEA methods. Therefore, it is necessary to conduct a thorough comparison of various DEA approaches under small biological replications. Here, we compare 6 performance metrics on both simulated and real scRNA-seq datasets to assess the adaptability of 8 DEA approaches, with a particular emphasis on how well they function under small biological replications. Our findings suggest that DEA algorithms extended from bulk RNA-seq are still competitive under small biological replicate conditions, whereas the newly developed method DEF-scRNA-seq which is based on information entropy offers significant advantages. Our research not only provides appropriate suggestions for selecting DEA methods under different conditions, but also emphasizes the application value of machine learning algorithms in this field.

## 1. Introduction

Human Genome Project in 1980s unveils the genomics research prologue [1], propelling population genomic analysis to unprecedented heights. Tang et al. (2009) proposed single-cell RNA sequencing (scRNA-seq) technology [2], which not only allows for the study of gene expression status at the single-cell level, but also solves the problem of cell heterogeneity that traditional genomic analysis cannot explain. Since then, genomics research has entered the era of single cell biology, which is of great significance to the study of the regulation mechanism of gene expression during cell growth. Recently, with the rapid development of high-throughput sequencing technology, bioinformatics has entered the era of big data and massive sequencing data has gradually become one of the most important sources for discovering new cell types. As the critical step in single-cell data analysis, differential expression analysis (DEA) is often used to identify genes that are susceptible to structural changes in response to different exogenous factors and thus expressed in significantly different amounts between two or more conditions. These genes serve as an important reference point for investigating biological heterogeneity issues and lays the foundation for other secondary analyses such as gene set enrichment analysis, pathway analysis, and network analysis, among others. This technology has been widely used in immunology [3], oncology [4,5], and embryonic stem cells [6,7] to reveal gene regulation processes in both human embryonic development and disease treatments. Taking cancer as an example, Kim et al. (2015) compared gene expression profiles of tumor cells before and after treatment to detect differentially expressed (DE) genes and reveal the mechanism of tumor drug resistance, thereby providing ideas for clinical tumor treatment [4]. In the field of immunology, this technology can track the course of immune response and detect small changes in the composition and structure of immune cells. Mogilenko et al. (2021) combined single-cell transcriptomics and immunomics sequencing results to elaborate the mechanism of immune senescence and identify its cellular markers [8], making it possible to delay immune senescence and enhance human immunologic function. Wauters et al. (2021) initially constructed the pathogenesis of SARS-CoV-2 and presented the human immune response profile at the cellular level by analyzing transcriptomic data from alveolar lavage fluid of patients with mild and severe COVID-19, as well as healthy people [9], laying the groundwork for the development of targeted therapy. Besides, Mailem et al. (2022) studied the whole blood transcriptome dataset from COVID-19 and other diseases using scRNA-seq analysis technology (e.g., DE Analysis, Network Construction, GO Enrichment and KEGG Pathway Analysis), with a focus on the genetic similarity of these diseases in biology [10]. Finally, hub genes within highly preserved modules were detected, and possible reusable drugs were identified accordingly, which propels drug design to more effective post-COVID-19 therapeutics. As a result, detecting potential DE genes from massive sequencing data has emerged as a current research hotspot with significant application value.

Unlike traditional bulk sequencing, scRNA-seq data has some peculiar characteristics such as high sparsity, high-level noise, high heterogeneity, and high dimension [11], putting greater demands on the applicability of DEA methods. Currently, most DEA methods for single cell data usually construct hypothesis testing models for genes in different cells and determine whether these genes express differently across cell types based on generated gene-cell expression matrix. Although different genes may have synergistic or antagonistic effects in practice [12], early DEA methods (e.g., t-test, Wilcoxon rank sum test) usually neglect such interaction effect that may exist across genes and thus modeled separately. Take the Wilcoxon method as an example, as one of the most common non-parametric tests in statistics, it can verify whether the genes express differently between groups by calculating the probability that samples from different groups come from the same distribution. However, with the exponential growth of data volume, such methods have major limitations in both statistical and computational efficiency, so researchers developed the Bioconductor package [13], which encapsulates multiple object components into a single instance through S4 system. It can not only maintain the consistency of numerical data and metadata during the analytical process, but greatly reduce data storage space. Up to now, the platform has assembled dozens of different DEA methods, some of them can borrow count information across genes while modeling, which definitely make the results more accurate and scientific.

## 2.Overview of current DEA methods

According to the above contents, a selection of available DEA methods which can be used in single-cell data analysis are listed in Table 1. It compares various DEA methods based on their original data motivation, distributional assumptions, statistical models, and test statistics, and arranges them in the chronological sequence in which they were first proposed. In this paper, the available DEA approaches are first classified into three major categories (general approaches, bulk RNA-seq methods and scRNA-seq methods) based on their origin.

**Table 1:** Comparative overview of some DEA methods for scRNA-seq data.

Categories	Subcategories	Methods	Year	Distribution*	Model*	DE Test Stat.*	Reference
General Approaches	Parametric	t-test	1908	Norm.	T-test	T stat.	-
	Non- Parametric	Wilcoxon	1945	NP	NP	Wilcox test	-
Extended from bulk RNA-seq	Parametric	baySeq	2010	NB	Bayesian	Posterior prob.	[14]
		DEGSeq	2010	Poisson	MARS	Z-score	[15]
		Cuffdiff	2013	Beta-NB	MCP	Fold Change	[16]
		Limma	2014	Log-Norm.	LM	Bayesian Wald	[17]
		DESeq2	2015	NB	GLM	Wald	[18]
	Non- Parametric	NOISeq	2011	NP	NP	Count Change	[19]
		SAMSeq	2013	NP	NP	T stat.	[20]
Special for scRNA-seq	Parametric	MAST	2015	ZI	Hurdle Model	LRT	[21]
		Monocle2	2017	NB	GAM	LRT	[22]
		DESingle	2018	ZINB	TCP	LRT	[23]
		ZIAQ	2020	LR	MM	Fisher’s test	[24]
		Tweedieverse	2022	ZITweedie	GLM	Wald	[25]
		IDEAS	2022	ZINB	TCP	J-S Divergence	[26]
	Non- Parametric	SAMstrt	2013	NP	NP	T stat.	[27]
		D3E	2016	NP	NP	KS test	[28]
		ROSeq	2021	NP	NP	Wald	[29]
	Extended from ML Methods	DEF-scRNA-seq	2019	ZI	DEF	T stat.	[30]
		MRF-scRNA-seq	2021	ZINB	MRF	DEF Stat.	[31]
		scDEA	2022	ZI	Hybrid Model	Lancaster’s test (Chi)	[32]

* Norm: Normal distribution; NP: Non-Parametric; NB: Negative Binomial distribution; Log-Norm: Logarithmic Normal distribution; ZI: Zero Inflated distribution; ZINB: Zero Inflated Negative Binomial distribution; LR: Logistic Regression; ZITweedie: Zero Inflated Tweedie distribution; MARS: Multivariate Adaptive Regression Splines; MCP: Multiple-class Parametric; LM: Linear Model; GLM: Generalized Linear Model; GAM: Generalized Addictive Model; TCP: Two-class Parametric; MM: Mixed Model; DEF: Differential Entropy-like Function Model; MRF: Markov Random Field Model; LRT: Likelihood Ratio Test; J-S Divergence: Jensen-Shannon Divergence; KS test: Kolmogorov-Smirnov’s test.

Though Table 1 shows many usable DEA tools, their applicable scenarios may vary a lot accordingly. In practical applications, we may choose DEA methods based on the nature of origin data. Generally, non-parametric methods can be used to handle discrete read counts while parametric methods are usually specialized for continuous or normalized counts, and specific DEA methods can be further selected based on the assumption of data distribution. Therefore, in the following parts, instead of individually going over all methods listed above, we discuss their advantages and disadvantages based on the common categories.

### 2.1. General approaches

The first category is general approaches, mainly including two-sample t-test and Wilcoxon test. These methods are based on hypothesis testing models and compute test statistics using distance-like metrics across two conditions or cell groups. Although these methods can perform simple DE analysis, there exists several limitations. For example, these methods typically perform comparisons of two cellular group, however multi-group comparisons with lower statistical power are out of scope.

### 2.2. Bulk RNA-seq methods

The second category in Table 1 includes methods originally designed for bulk RNA-seq but later extended to scRNA-seq. These methods were initially used only for analyzing the average expression profile of cell populations in tissues, and they can also be used for single-cell differential analysis with appropriate refinements, though they mostly only allow differential gene mining between two groups. Further, bulk RNA-seq methods can also be divided into parametric and non-parametric methods. The former class usually make strict distributional assumptions of sequencing data and detect DE genes based on hypothesis testing. For instance, baySeq [14] and DESeq2 [18] assume that read counts are obtained from NB distribution and conduct bayesian model or generalized linear model based on the scRNA-seq data, Limma assumes that sequencing data follow log-normalization and perform an improved T-test based on linear model and empirical Bayes [17,33]. However, if the true distribution of data does not conform to the underlying assumptions, non-parametric methods can be better alternatives to their parametric counterparts. Such methods are usually distribution-free, they conduct hypothesis tests and detect DE genes by building a non-parametric model and estimating the parameters that can quantify the statistics based on the ranked list and make comparisons between two groups. NOISeq [19], SAMSeq [20], and other tools fall into this category. However, these methods may have limitations when applied to scRNA-seq, which can only give conclusions under the average expression of cell populations rather than single cell.

### 2.3. scRNA-seq methods

With the increasing use of second-generation sequencing technology and the advancement of genomics research, the study of gene regulation in organisms has become the focus of biologists’ attention, while the traditional bulk sequencing method is evidently challenging to meet these demands. Thus, the third category of methods exclusively developed for single-cell sequencing are proposed, which are more targeted and can not only analyze gene expression in individual cells, but also detect biomarkers across cell types. As presented in Table 1, these methods can also be classified into parametric and non-parametric based on distributional assumptions of read counts. Based on the statistical models used in the algorithm, Das et al. (2022) further subdivided parametric methods into five minor categories: generalized linear, generalized additive, hurdle, mixture models and two-class parametric approaches [34]. Among them, generalized linear models (GLM) assume that expression counts follow an exponential family distribution, and gene expression counts are usually related to linear predictors through a non-linear link function. By estimating unknown parameters and calculating values of likelihood ratio test (LRT) statistics, these methods can determine whether certain genes express differently or not across cell groups. Specifically, methods like DECENT [35] and Tweedieverse [25] fall into this category. Generalized additive models (GAM) are natural extensions of generalized linear models which use additive smooth spline functions to capture the relationship between individual cells and gene expression. Like GLM models, GAM detect DE genes based on LRT or Wald statistics. Methods belonging to GAM class includes Monocle [36], Monocle2 [22], and tradeSeq [37], etc. Hurdle models usually assume that expression counts follow a zero-inflated distribution and establish linear models separately for zero and non-zero counts. MAST [21] is a typical example of method from this category. Mixture models (MM) assume that gene expression counts are derived from combinations of different distributions, such as the Beta-Poisson distribution, the Poisson-NB distribution, and so on. By utilizing the generalized linear model framework and aggregating their results, such methods can compute the P-values for each gene and detect DE genes among. The MM class is more common and includes methods like SCDE [38], ZIAQ [24], etc. Compared with the above DEA approaches, two-class parametric models (TCP) are more straight forward and do not require complicated linear models. Similar to t-test, TCP models test the estimated value of mean parameter across the two groups rather than on each individual genes, which are simpler and faster compared to other categories of methods. However, such methods have obvious limitations in that they cannot be generalized to multiple cellular groups and are more sensitive to sparsity or dropout. This category includes methods such as DESingle [23] and IDEAS [26].

Although there are now many effective techniques for DE analysis, currently the detection of DE genes still remains a challenge. Firstly, the DE genes discovered by various DEA methods differs from each other and the results depend on their underlying data structures. Secondly, single-cell detection technologies that incorporate much histology are quickly developing, requiring advanced techniques for DE analysis that traditional DEA approaches cannot effectively utilize. Thirdly, with the dimension of sequencing data getting larger, we must take the speed of computational processing into consideration. To conquer these difficulties, more and more machine learning algorithms have been gradually introduced into scRNA-seq methods in recent years. For example, Wang et al. (2019) first introduced the theory of information entropy into scRNA-seq field and proposed a DEA method based on differential entropy-like function (DEF) [30], which reflects gene’s differential expression level by calculating value of differential entropy-like function between groups and is proved to be well suited for low replicates, zero counts and multiple conditions. Li et al. (2021) developed a markov random field model called MRF-scRNA-seq for network‑based DE analysis [31], which can capture gene network and take gene dependencies among cells into consideration. By using Expectation-Maximization algorithm, model parameters can be easily estimated and then DE status of each gene can be inferred. Li et al. (2022) proposed scDEA [32], an ensemble learning based DEA method that can greatly reduce the contingency of results by integrating P-values obtained from several individual DEA methods for each gene, which is proved to produce a more stable result under different experimental settings. Compared with traditional DEA methods, the above new methods based on machine learning algorithms are more data-adaptive, less affected by data noise or dropouts and typically produce more stable and accurate result.

### 2.4. Related challenges

Although many newly proposed DEA methods are available in the literatures during the recent years, up to now there is still no consensus on the applicability of different DEA methods under specific experimental conditions, so a comprehensive comparison of these methods and appropriate guidelines for selecting proper approaches that can perform better under specific experimental settings are required. Many relevant studies have also been carried out in recent years, for example, Soneson et al. (2013) discovered that non-parametric methods have a better performance under large biological replicate conditions by evaluating 11 widely used DEA methods on simulated datasets [39], but the algorithms evaluated in this paper were limited to bulk RNA-seq methods. Zhang et al. (2014) compared three commonly used software tools, and pointed out that edgeR performs better in identifying real DE genes, while DESeq is advised if there is a higher concern on false positive issues [40]. Das et al. (2021) evaluated 19 widely used DEA methods on different datasets and indicated that DECENT and EBSeq performs better for scRNA-seq data [41]. However, these existing studies omitted DEA methods extended by machine learning algorithms, and most of the empirical research was conducted under large biological replications, research on expression counts with a small number of biological replicates is still scarce.

However, biological replicates, which means to use different biological samples under the same conditions to measure biological differences between samples, are absolutely necessary in scRNA-seq experiments, especially in DEA process. This is because there exists a series of environmental factors that may produce individual differences in organisms. As our sequencing experiments cannot cover all samples, it is vital to adopt biological repetitive experimental approaches to minimize the impact of such differences as much as possible. Obviously, the more biological replicates are involved in the sequencing experiments, the less our experiment will be affected by such individual differences and thus can obtain a better result. Compared with biological variability, technical variability, usually caused by random noise and sequencing error, could only reflect the reproducibility of an assay rather than the phenomenon in the research, which is much smaller under current scRNA sequencing technology [42]. For example, Blainey et al. (2014) demonstrated the relationship between biological replicates and technical replicates by measuring the expression level of genes in mouse liver cells. They found that the biological replicates of cells can better reflect the overall population than the technical replicates, indicating that sequencing resources should be better biased towards biological replicates [43]. Bell (2016) indicated that though different values are given by conducting technical repeating experiments, the p-values given is not rational [44]. So, it may be useless to inflate sample sizes by simply increase technical replicates rather than biological replicates. However, obtaining enough biological replicates is far more difficult than it in technical replicates. Even though bioinformatics is progressing toward larger sample sizes, scRNA-seq experiments are still too expensive to obtain sufficient biological replicates, therefore small biological replicates will remain a universal feature of RNA-seq experiments for a long time [39]. As a result, it is critical to investigate the applicability of different DEA methods under small biological replications.

**Fig 1:**
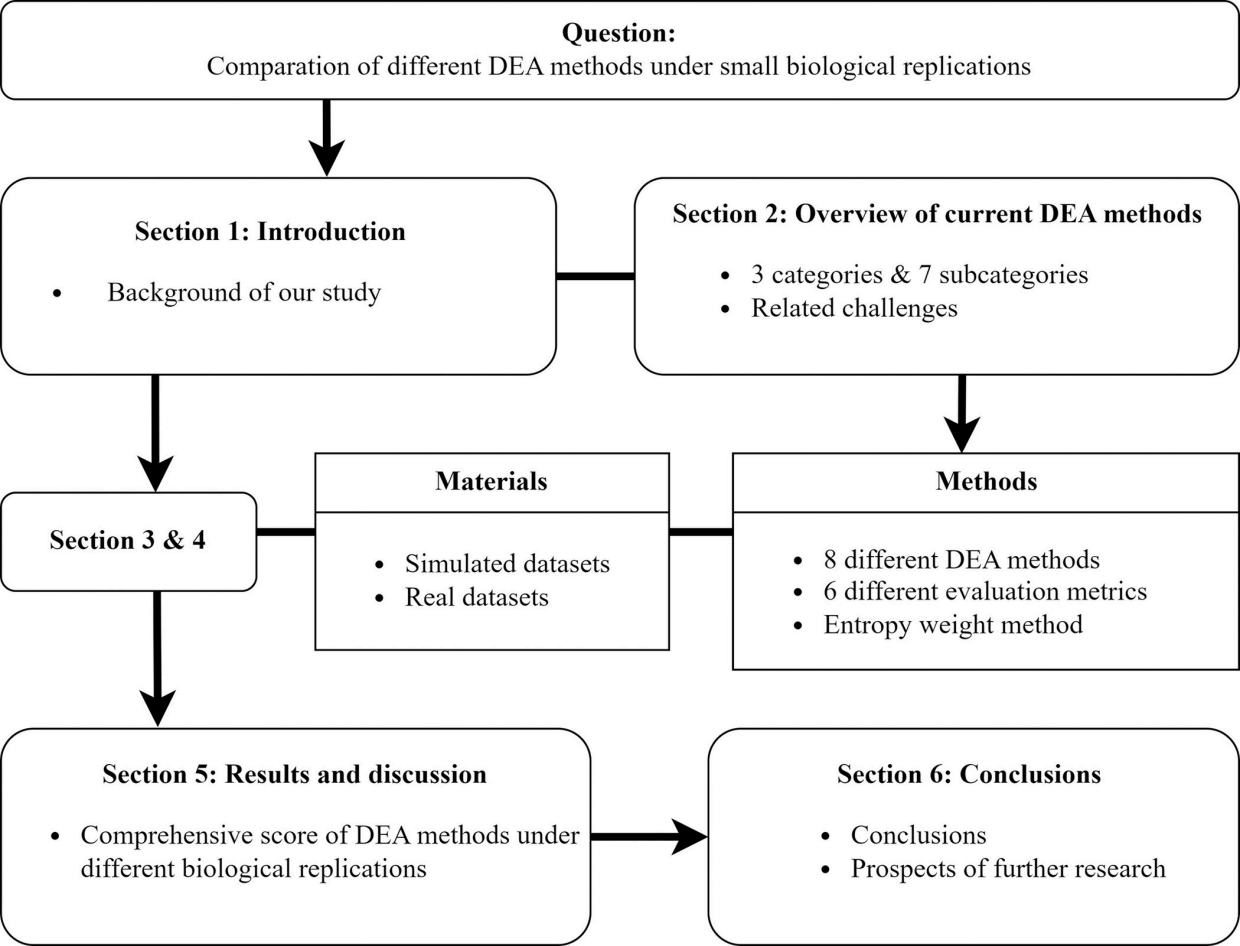
Research framework of this paper.

We, therefore, aim to conduct a comprehensive comparison of different DEA methods and primarily focus on the performance of each method in the case of a small number of biological replicates (*K*<6) to provide a guideline for selecting proper DEA methods under different biological replications. According to [41], performance of different DEA methods varies greatly, and it may depend on their underlying models, DE test statistic, and data characteristics, etc. In order to cover as many types of DEA methods as possible, we consider all 7 subcategories of DEA methods mentioned in the previous paper and select DEA methods based on their distributional assumptions, statistical models, and test statistics. Finally, the following 8 DEA methods were used in our experiment: (i) general DEA methods, including parametric method T-test and non-parametric method Wilcoxon; (ii) methods originally designed for bulk RNA-seq but later extended to scRNA-seq, including parametric method DESeq2 and Limma-voom, and non-parametric method SAMSeq; (iii) methods exclusively developed for single-cell sequencing, including parametric method DEsingle, non-parametric method ROSeq, and newly developed method DEF-scRNA-seq which is based on machine learning algorithm. The paper is organized as follows: Section 1 introduces the background of our study. Section 2 provides an overview of current DEA approaches along with possible challenges that may exist. Section 3 provides materials involved in our research, including simulated datasets and real datasets. Section 4 introduces DEA methods and evaluation metrics used in our experiment. Section 5 presents the results and discussion of our research on both simulated and real scRNA-seq datasets. Section 6 presents conclusions and prospects. The framework of this paper is shown in Fig 1.

## 3. Materials

**Notations:** *y*_*ij*_: expression counts of *i*th (*i* = 1,2,⋯,*m*) gene in *j*th (*j* = 1,2,⋯,*n*) cell; *Y*: design matrix for cell group information, whose *i*th row is *Y*_*i*_ = [*Y*_*i*1_,*Y*_*i*2_,⋯,*Y*_*in*_]; *μ*: mean expression of original counts for *i*th gene; *σ*^2^: variance of original counts for *i*th gene.

### 3.1. Simulated datasets

Since we do not have access to true DE genes in the real RNA-seq dataset, we began our research by comparing and evaluating different DEA methods with simulated datasets. In order to make the simulation experimental results more reliable, the sparsity feature of sequencing data must be taken into account while generating simulated datasets. Currently, Negative Binomial (NB) distribution has been used in the majority of studies to create simulated data [39]. With the understanding of sequencing principles gradually improved, the theoretical distribution of scRNA-seq data has been constantly updated in recent years, and the simulation approach can be upgraded. Thus, we summarize and compare the theoretical distribution of sequencing data first.

#### 3.1.1. Theoretical distribution of scRNA-seq data

**Fig 2:**
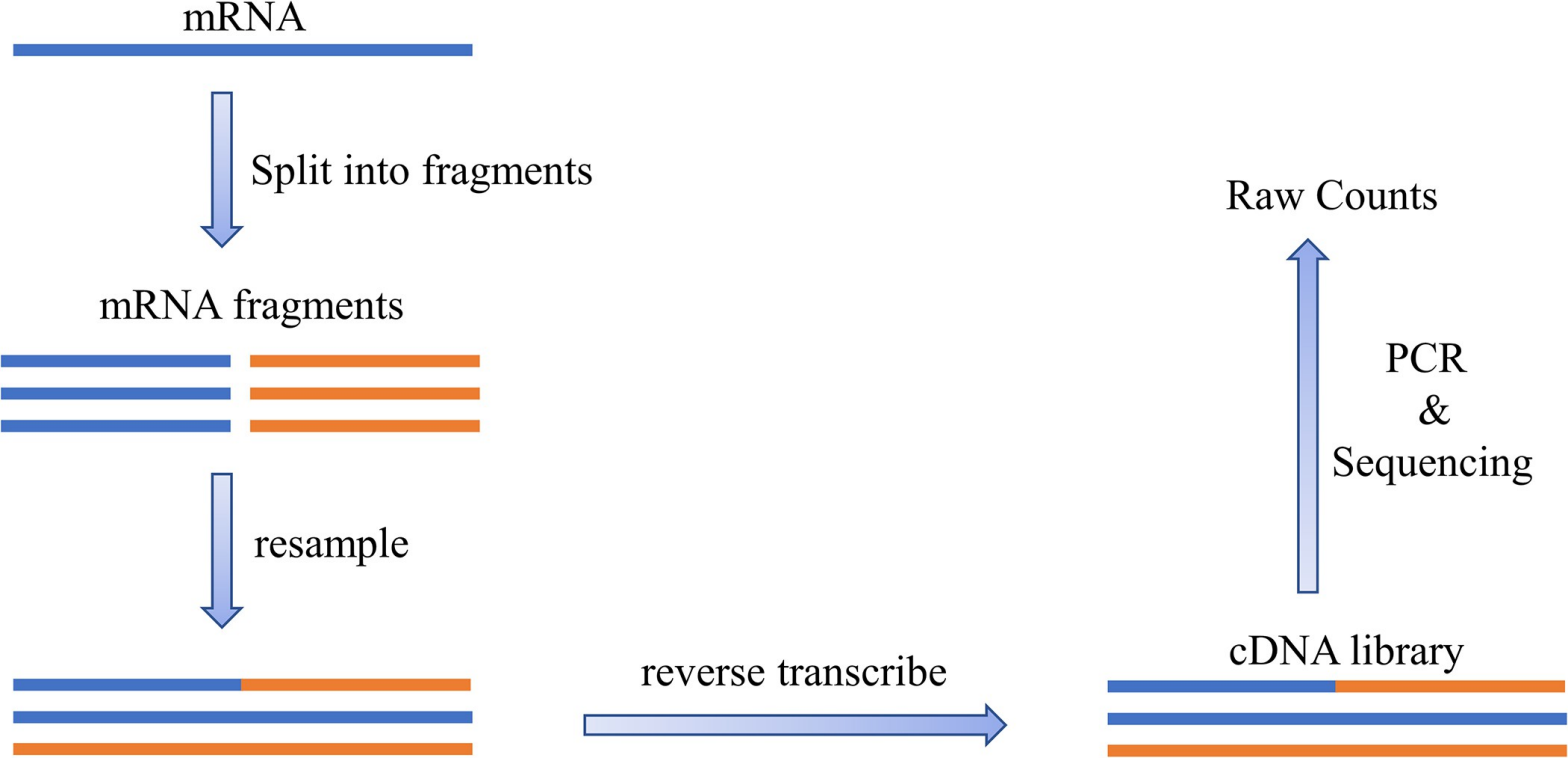
Schematic of RNA sequencing principle.

The fundamental process of RNA sequencing is shown in Fig 2. It first isolates mRNA and splits them into small pieces, then mRNA fragments are spliced by resampling and are reverse transcribed into cDNA libraries. By sequencing the cDNA, we can determine the order of different bases which are remained on the single-stranded RNA and obtain the count of reads positioned to a specific reference gene, which accurately reflects the expression level of different genes.

Based on all above processes, researchers have proposed various theoretical distributions of RNA-sequencing data, which provides convenience for simulation and analysis of transcriptome data. The following are the most commonly used distributions:

(1) Poisson distribution

Assume $\theta_{ij}(i=1,2,\cdots ,m;j=1,2,\cdots ,n)$ is the read count of *i*th mRNA in sample *j*, *l*_*i*_ is the length of *i*th mRNA. Since all sites of mRNA may be used as the starting position of a sequence to generate a reading segment, the probability of a sequence segment generated from *i*th mRNA in sample *j* is:

$$\pi_{ij}=\frac{\theta_{ij}l_{i}}{\sum_{i=1}^{m}\theta_{ij}l_{i}}$$ (1)

Jiang et al. (2009) indicated that resample of mRNA fragments in sequencing process can be abstracted into a simple random sampling process [45], which means each segment is an independent sample from sample *j*. Under this assumption, we can assume that the original read count of each sequence follows binomial distribution. Considering that the number of genes in single-cell sequencing is large (usually varying from thousands to tens of thousands), and the number of mRNA fragments in sequencing library is large too, then the probability of each mRNA segment being selected convergence to zero. According to Poisson Theorem, the original read count can be approximated to follow Poisson distribution:

$$y_{ij}∼P(\lambda_{ij})$$ (2)

where *λ*_*it*_ = *y*_*ij*_*π*_*ij*_ is the parameter of Poisson distribution and the mean and variance of read counts are both *λ*_*ij*_.

**Fig 3:**
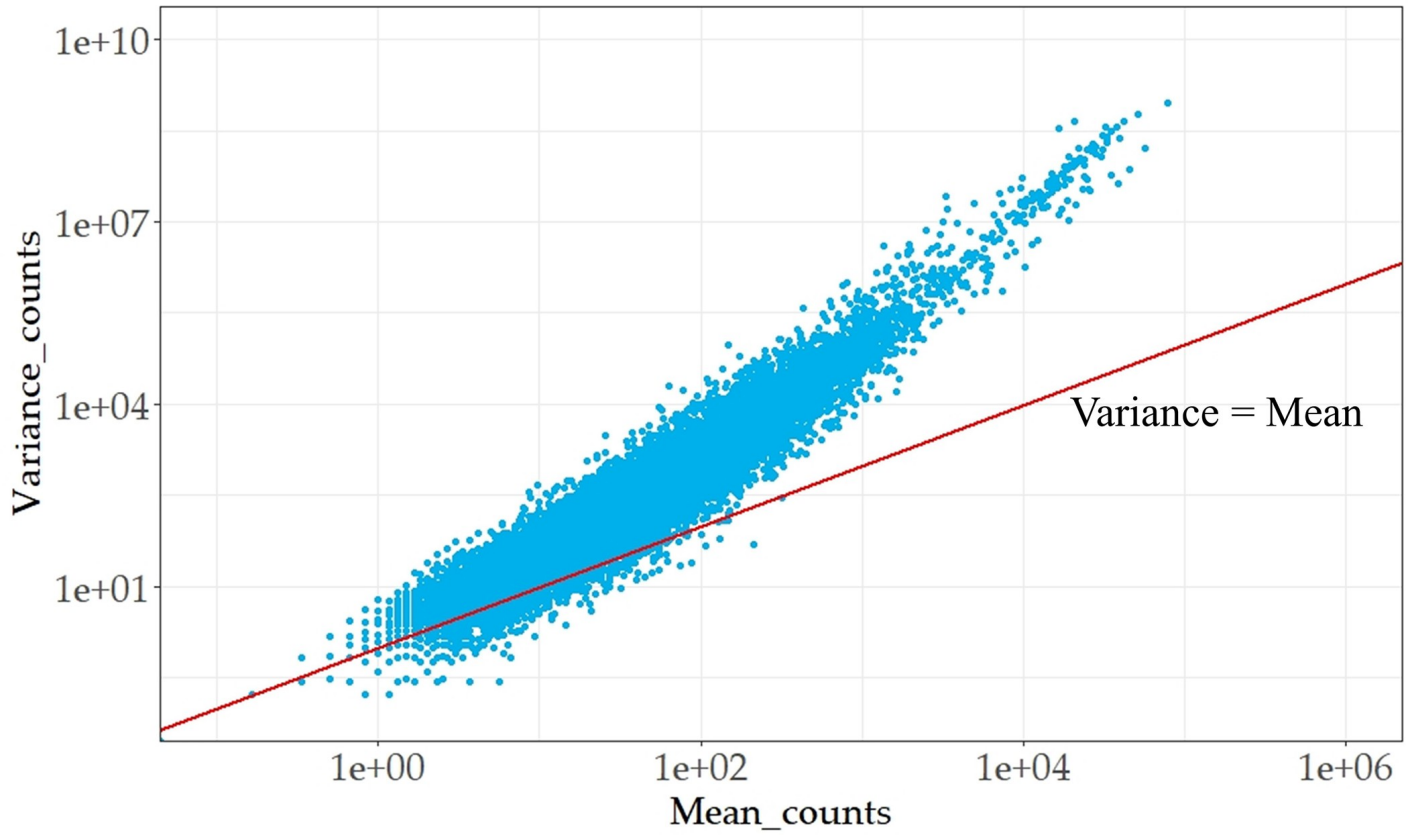
Mean-variance relationship of real scRNA-seq data.

However, descriptive statistics of real sequencing data shows that read count variance is significantly greater than its mean expression (Fig 3). The result does not conform to Poisson distribution whose mean value is always equal to its variance. This is because scattering noise affects the resample process of mRNA fragments during sequencing, making it differ from simple random sampling process and thus resulting in a deviation of resample process from previous assumptions. Only when the number of biological replicates in sequencing experiment is large enough that the randomness brought by samples is sufficient to compensate for the incomplete randomness of mRNA fragment resampling, can the sequencing data be approximated as following Poisson distribution, then variance of original read counts is also close to its mean value. However, due to high economic costs, the number of biological replicates in the experiment is difficult to meet such requirements, thus the assumption of Poisson distribution has major limitations in practice.

(2) Negative Binomial distribution

To explain the phenomenon of high variance in sequencing data, Robinson et al. (2010) described the original read counts of scRNA-seq data as an over-discrete Poisson distribution and proposed Negative Binomial distribution instead [46], then:

$$y_{ij}∼NB(M_{i}p_{ij},\Phi_{i})$$ (3)

where *M*_*i*_ means sequencing library size; *p*_*ij*_ is the relative abundance of gene *i* in sample *j*; Φ_*i*_ is a dispersion parameter, which represents the degree of biological variation between samples. Robinson suggested that expected mean and variance of original read counts obtained by sequencing satisfy Eqs (4) and (5):

$$\mu =M_{i}p_{ij}$$ (4)

$$\sigma^{2}=\mu +\Phi_{i}\mu^{2}$$ (5)

Since Φ_*i*_ is a non-negative constant, *σ*^2^>*μ* is always correct, which means under the assumptions of NB distribution, the variance of read count is indeed greater than its mean expression. Only when Φ_*i*_→0, the NB distribution degenerates to Poisson distribution where *σ*^2^ = *μ*.

(3) Tweedie distribution

Although the Negative Binomial distribution takes the overdispersion property of sequencing data into account and has been used in many popular DEA methods like DESeq2 and edgeR, on the other hand, RNA-seq data is typically zero-inflated, which means our gene-cell expression matrix is a highly sparse matrix with a large percentage of zero counts. Therefore, it is often difficult to accurately describe real sequencing data with a NB distribution. Accordingly, Esnaola et al. (2013) proposed that the Poisson-Tweedie family of distributions can be used to appropriately simulate RNA-seq data [47], which can better restore the zero-inflated feature of RNA-seq data and provide a new idea for data simulation and modeling:

$$f_{Y}\left(y|\theta ,\Phi \right)=a\left(y,\Phi \right)\cdot \exp\{\frac{1}{\Phi }(y\frac{\mu^{1-\rho }}{1-\rho }-\frac{\mu^{2-\rho }}{2-\rho })\}$$ (6)

where *μ* is the mean value of original read counts, which portrays the average gene expression level; Φ is a dispersion parameter, which represents the degree of biological variation between samples; *ρ* is an indicator parameter, which determines the specific distribution of variable *Y*. When *ρ* = 1, *Y* degenerates to Poisson distribution; when *ρ* = 2, *Y* degenerates to Gamma distribution; Only when 1<*ρ*< 2, *Y* follows Tweedie distribution, which is also called Poisson-Gamma mixture distribution.

(4) Comparation of three distributions

As the understanding of sequencing principles has gradually improved, the theoretical distribution of scRNA-seq data has been constantly updated in recent years. Based on the theoretical introduction of three popular data distribution assumptions mentioned above, the main features, advantages, and disadvantages of each distribution are summarized in Table 2. With the development of Next-generation sequencing technology, the dimensionality of sequencing data is increasing rapidly, making the zero-inflated feature of RNA-seq data non-negligible, where traditional models are no longer effective. The proportion of Tweedie distribution family provides a new idea for the simulation and modeling of RNA-seq data.

**Table 2:** Comparison of the three data distributions.

Distribution	Parameters	Advantages	Disadvantages
Poisson distribution	*λ*_*ij*_	Approximating the sampling process of mRNA fragments in sequencing as "simple random sampling process" to simplify the complexity in analyzing	The Poisson distribution where sample mean equals to its variance cannot objectively describe the sample dispersion brought about by biological replication
Negative Binomial distribution	*μ*、*σ*^2^	Use an over-discrete Poisson distribution instead to adapt overdispersion property of sequencing data	The distribution cannot accurately portray the zero-inflated features and trailing phenomenon of RNA-seq data
Tweedie distribution	*μ*、Φ、*ρ*	The introduction of three-parameter distribution can better restore the trailing features of sequencing data, and Tweedie distribution can generate zero values with a certain probability, which is consistent with zero-inflated feature of RNA-seq data	Interpretability of simulated frequencies for zero expression values needs to be enhanced

#### 3.1.2 Data synthesis scheme

According to Table 2, in order to recreate the zero-inflated and data trailing features that may exist in real scRNA-seq datasets, we generated simulated datasets based on the Tweedie distribution. Usually data simulation of scRNA-seq needs to consider two or more experimental groups, *K* biological replicates, and the expression counts of DE/non-DE genes under different conditions.

In this paper, only two experimental conditions were considered, which means all samples are divided into experimental and control groups. Schurch et al. (2016) indicated that in practical DE analysis, at least six sets of biological replicate experiments should be carried out in order to detect DE genes as many as possible with a low value of false positive rate [48]. However, scRNA-seq experiments are often too expensive to get sufficient replications, so it is necessary to evaluate whether current DEA methods can be used in small biological replications. Since we primarily concerned with the performance of different DEA methods under small biological replicates, our empirical analysis was limited to biological replications of 2 to 5. Given that DE genes generally account for 5% ~ 10% of the total number of genes in practice, we randomly generated 10,000 read counts across two groups, including 9,000 non-DE genes, 500 up-regulated DE Genes (DE genes whose expression counts increase significantly in experimental groups) and 500 down-regulated DE Genes (DE genes whose expression counts decrease significantly in experimental groups). The simulation data generation scheme for different genes is shown in Table 3. We control the expression of genes by adjusting the value of parameter *μ* and Φ:for non-DE genes, we assign the same value of all parameters to the experimental group and control group, ensuring that all data comes from the same distribution (Poisson-Gamma mixture distribution); for up-regulated DE genes, we raise the values of *μ* and Φ to increase the mean and variance of the overall distribution, resulting in higher expression levels of genes in the control group; while for down-regulated DE genes, we decrease the values of parameters *μ* and Φ.

**Table 3:** The simulation data generation scheme for different genes.

Gene Types	Experimental groups	Control groups
Non-DE Genes	$\begin{array}{l} \rho =1.2\\ \mu =20\\ \Phi =2 \end{array}$	$\begin{array}{l} \rho =1.2\\ \mu =20\\ \Phi =2 \end{array}$
Up-regulated DE Genes	$\begin{array}{l} \rho =1.2\\ \mu =20\\ \Phi =2 \end{array}$	$\begin{array}{l} \rho =1.2\\ \mu =40\\ \Phi =2.2 \end{array}$
Down-regulated DE Genes	$\begin{array}{l} \rho =1.2\\ \mu =20\\ \Phi =2 \end{array}$	$\begin{array}{l} \rho =1.2\\ \mu =10\\ \Phi =1.8 \end{array}$

**Fig 4:**
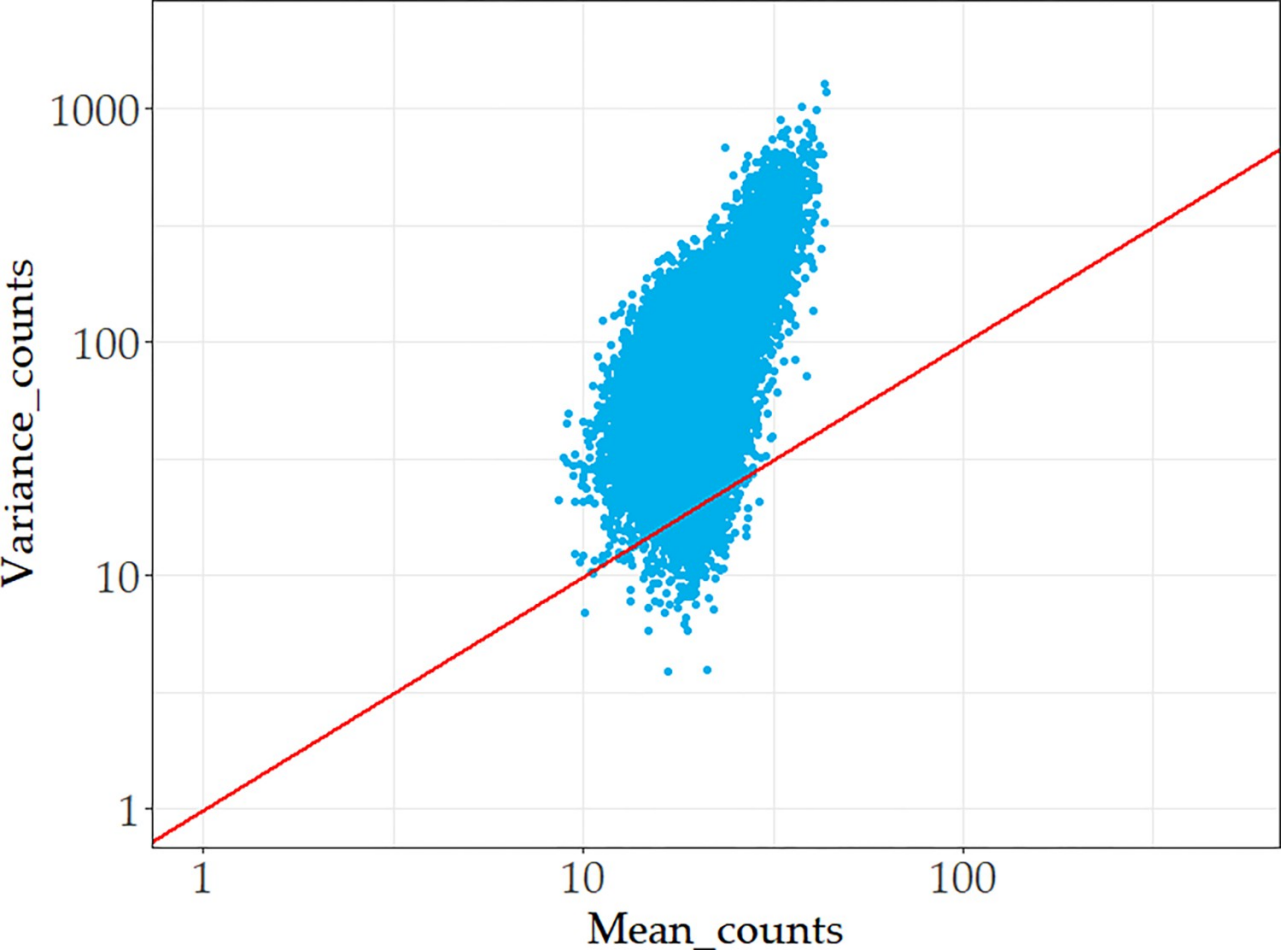
Mean-variance relationship of simulation dataset.

To further verify how well the generated simulation dataset can replicate the characteristics of real scRNA-seq data, we performed a descriptive statistical analysis on the generated simulation dataset (Fig 4) and compared the results with the real sequencing data shown in Fig 3. We discovered that the two datasets have similar distributional features whose variance is greater than its mean value, thus we believe the data synthesis process was appropriate.

Since the generation of simulated data is random and the conclusion from a single analysis is not statistically significant, the above process was repeated 30 times for each experimental condition, and the average of the 30 analyses was used as the result to identify the advantages and drawbacks of each DEA methods.

### 3.2. Real scRNA-seq dataset

To further assess the performance of the above DEA approaches, we use two real scRNA-seq datasets from GEO (https://www.ncbi.nlm.nih.gov/geo/)–a public functional genomics data repository.

#### 3.2.1 GSE150861

This real scRNA-seq dataset was downloaded with accession number GSE150861. It contains single-cell transcriptome data of peripheral blood mononuclear cells (PBMCs) from severe COVID-19 patients, with a total of 33538 genes and 15420 cell samples. All samples were divided into two groups: experimental (8468 cell samples from patients in remission after tocilizumab treatment) and control (6952 cell samples from patients with severe disease who were not receiving treatment). Each experimental condition received three biological replicates.

#### 3.2.2 GSE235436

This real dataset was downloaded with accession number GSE235436. It contains expression profiling of COVID-19 patients obtained by high throughput sequencing, with a total of 33538 genes and 4451 cell samples.

## 4. Methods

### 4.1. Pre-processing steps

**Fig 5:**
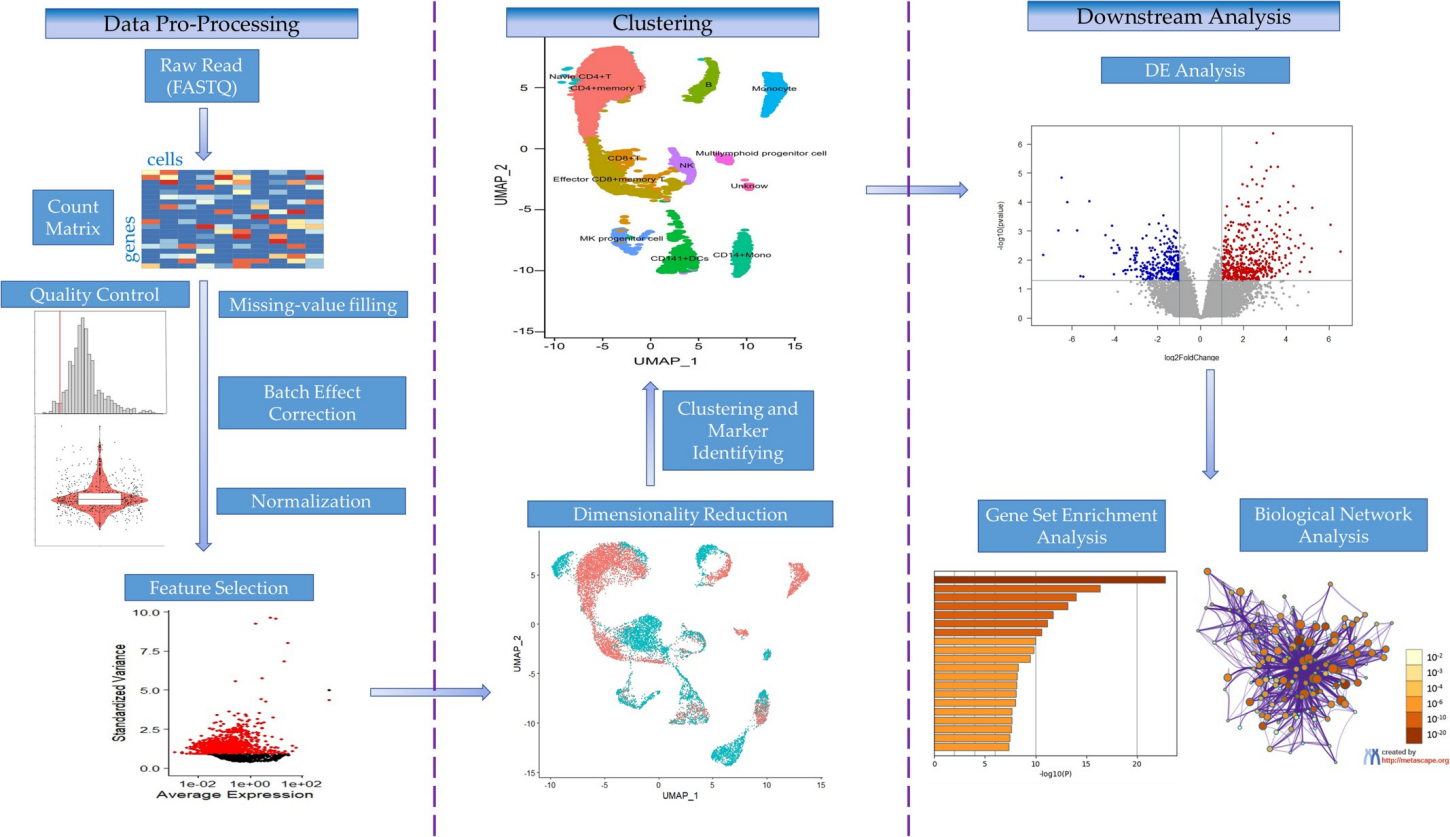
Full flow of scRNA-seq data analysis.

Generally, the DEA process starts with expression matrix *Y* whose *i*th row is *Y*_*i*_ = [*Y*_*i*1_,*Y*_*i*2_,⋯,*Y*_*in*_], where *Y*_*i*j_ represents expression counts of *i*th gene in *j*th cell sample. However, such matrix count cannot be directly used for DE analysis and a series of pre-processing steps are needed before conducting DE analysis. The entire flow of scRNA-seq data analysis is shown in Fig 5.

The first step in processing scRNA-seq data is to exclude cell barcodes that cannot represent complete individual cells. By calculating the gene expression status on each cell sample with the help of Quality Control (QC) plot, we filter out dead cells, broken-membranes cells, or doublets cells. Secondly, a normalization step must be applied to remove the interference of sequencing depth during subsequent analysis, as the amount of RNA per cell can vary greatly between cells. Log-normalization method, for example, is a widely used normalization methods, which not only reduces the skewness of sequencing data, but also preserves zero expression values in the original sparse matrix. Next, to obtaining true biological signal, a batch effect correlation or data integration step should be taken into consideration with the help of R package like Combat or mnnCorrect [49]. Recently, Korsunsky et al. (2019) developed Harmony [50], which is a prominent approach for data integration with superior performance and fewer computational resources, especially when datasets are assayed with different technologies. Among all these methods, Luecken et al. (2022) present a benchmarking study of data integration methods in complex integration tasks, which can help identify optimal data integration methods for new data [51]. Fourthly, in order to escape “curse of dimensionality” problem, a feature selection and data dimensionality reduction process is needed. By using Principal Component Analysis (PCA) to compress redundant information and then use Uniform Manifold Approximation and Projection (UMAP) to find a low-dimensional representations that maintains adjacency in a high-dimensional space, we can visualize sequencing samples and lay the foundation for cell clustering. By clustering cell samples with similar expression profiles, we can visualize the whole expression status. As we can see, many different methods can be applied in every step and may lead to different results. Therefore, it is necessary to conduct benchmarking analyses, which can compare their performances and help choose best tools according to the data types and complexity.

### 4.2. DEA methods

In the following part, we give a brief overview of DEA methods used in our comparation experiment. Usually, a DE analysis begins with a processed expression matrix having different samples as variables and different genes as statistical units, by which we can compare distributions of expression levels among cell samples. Here we consider all 3 categories of DEA methods introduced in Section 2 and select DEA methods based on their distributional assumptions and statistical models. The principle of each DEA method is as follows.

#### 4.2.1. T-test

T-test is a traditional method for testing whether there is a difference in the mean expression of genes between two cellular groups and thus detecting potential DE genes. Traditional T-test require that data roughly follow normal distribution in order to produce accurate results. However, the presence of zero-inflated features causes scRNA-seq data to be highly right skewed, clearly violating the above requirements. As a result, the original expression matrix must be normalized before the T-test, using methods such as TMM normalization, log-normalization, and so on. The test statistic of T-test can be defined as:

$$t_{i}=\frac{\mu_{i1}-\mu_{i2}}{\sigma_{i}}$$ (7)

$$\sigma_{i}=\sqrt{\frac{{s_{i1}}^{2}}{N_{1}}+\frac{{s_{i2}}^{2}}{N_{2}}}$$ (8)

where *μ*_ij_、*s*_*ij*_^2^ are the mean and variance of normalized counts of *i*th gene across two groups; *N*_*i*_ is number of cell samples in each group; *t*_*i*_ is the test statistic of *i*th gene, we can say that the *i*th gene is differentially expressed across groups if *t*_*i*_ is greater than the critical value. In this paper, we used *t*.*test* function in R software to execute this method.

#### 4.2.2. Wilcoxon rank sum test

Wilcoxon rank sum test, also known as Mann-Whitney U test, is a popular non-parametric method in statistics. Its main idea is that if two samples come from the same distribution, there will be little difference in their ranks. Unlike T-test, this method does not require a specific data distribution and thus has a broader range of application. Therefore, we can apply this method to RNA-seq data to test whether the mean expression of a gene differs significantly between two groups. In this paper, we used *wilcox*.*test* function in R software to execute this method.

#### 4.2.3. DESeq2

DESeq2 is a DEA method based on generalized linear model [52], which assumes that *Y*_*ij*_ follow NB distribution (Eqs (9) and (10)) and uses moment estimation to impute mean and variance of read counts on each gene. According to the results of Eq (5), the variance in NB distribution increases with its mean expressions, so the absolute values of variance between different groups are not comparable. To address this issue, DESeq2 allows for weighted combination of variances by borrowing count information across remaining genes and then assessing differential gene expression information in multifactorial experiments using dispersion and fold change shrinkage, which makes the data more suitable for modeling. Further, a generalized linear model as in Eq (11) is developed for each gene separately, and the P-value of the Wald test or log2FoldChange is calculated.

$$Y_{ij}∼NB(s_{j}\mu_{ij},\Phi_{i})$$ (9)

$$s_{j}=\frac{Y_{ij}}{\sqrt[{n}]{\prod_{j=1}^{n_{A}}Y_{ij(A)}\cdot \prod_{j=1}^{n_{B}}Y_{ij(B)}}}$$ (10)

$$\log\mu_{ij}=\sum_{r}\beta_{ri}\cdot X_{jr}$$ (11)

where *μ*_*ij*_ is the expected read counts for gene *i* under condition *j* (In two-groups experiments, *j* = A or B), Φ_*i*_ is the scatter coefficient of read counts for gene *i*, *s*_*j*_ is the scale factor, *n*_*A*_ and *n*_*B*_ are the number of biological replicates in two sets of conditions, *X*_*jr*_ is the design matrix about the sample groups, *r* is a covariate factor, *β*_*ri*_ is parameter to be estimated for the GLM model, reflecting the logarithmic ratio of gene *i* to the covariate *r*. In this paper, we used *DEseq* function of DESeq2 R package to execute this method.

#### 4.2.4. Limma-voom

Limma is an improved T-test based on linear model and empirical Bayes [33]. It first serializes discrete read counts, then use linear models to fit the expression level of each gene and detect DE genes by an improved T-statistic. Limma’s core steps are primarily handled by two functions: Lmfit and eBays. The Lmfit step uses a linear model to fit the expression value of each gene (Eq (12)); eBays method uses the idea of empirical Bayes to compress the residual variance of gene expression toward its overall trend, yielding the posterior estimate of sample variance ${\tilde{s}}_{j}^{2}$ (Eq (13)). By using the posterior estimate statistics ${\tilde{s}}_{j}^{2}$ instead of *s*_*j*_^2^, we can obtain a more stable T-statistic improved by empirical Bayes (Eq (14)).

$$y_{i}=X\beta_{i}+b_{i}$$ (12)

$${\tilde{s}}_{j}^{2}=\frac{d_{0}{s_{0}}^{2}+d_{j}{s_{j}}^{2}}{d_{0}+d_{j}}$$ (13)

$${\tilde{t}}_{jk}=\frac{{\overset{⌢}{\beta }}_{jk}}{u_{jk}{\tilde{s}}_{j}}$$ (14)

where *d*_*j*_ is the error degrees of freedom for *j*th gene, *d*_0_ and *s*_0_ are priori estimates of *d*_*j*_ and *s*_*j*_, which also measures the additional information borrowed from the rest genes to infer each individual model. Only when *d*_0_ = 0, the ${\tilde{t}}_{jk}$ statistic degenerates to conventional T-statistic. In this paper, we used *lmFit* and *eBays* function of limma R package to execute this method.

#### 4.2.5. SAMSeq

SAMSeq is another popular non-parametric method in which the test statistic includes a regularized constant term *s*_0_ to the denominator of T-statistic. Though *s*_0_ is a very small positive number, it can effectively avoid classifying genes with small variances as DE genes, improving the stability of T-test and adapting better to data with smaller biological replicates. T-statistic obtained from SAMSeq was shown as follows:

$$t=\frac{{\bar{x}}_{A}(i)-{\bar{x}}_{B}(i)}{s_{0}+s(i)}$$ (15)

$$s(i)=\sqrt{\frac{1}{m+n-2}(\frac{1}{m}+\frac{1}{n})\{{\sum_{m}\left[x_{m}(i)-{\bar{x}}_{A}(i)\right]}^{2}+{\sum_{n}\left[x_{n}(i)-{\bar{x}}_{B}(i)\right]}^{2}\}}$$ (16)

where ${\bar{x}}_{A}(i)$ and ${\bar{x}}_{B}(i)$ are gene’s mean expressions of two groups, *m* and *n* are number of replicates of two groups, *s*(*i*) is gene specific divergence, which is an estimate of sample variance, and *s*_0_ is the regularization constant obtained by minimizing the overall variance. In this paper, we used *SAMseq* function of samr R package to execute this method.

#### 4.2.6. DEsingle

DEsingle is a DEA method designed specifically for scRNA-seq data that assumes read counts follow a zero-inflated negative binomial distribution. Different with other DEA methods, it can further divide the observed zero values into two categories including true zero counts and dropouts with the help of classified data and model them separately, allowing it to better adapt to the features of zero-inflated in sequencing data. By estimating the parameters of the two models and performing likelihood ratio tests, the final differential expression levels of genes can be obtained. In this paper, we used *DEsingle* function of DEsingle R package to execute this method.

#### 4.2.7. ROSeq

Similar to Wilcoxon rank sum test, ROSeq is another rank-based non-parametric DEA method. This algorithm first use TMM normalization to obtain normalized data and then serialize it with the help of voom transformation [29]. Following that, we can divide the data into different bins and rank each gene based on the sequential order of its expression range. To statistically test whether a gene is differentially expressed between two subpopulations, ROSeq uses an asymptotically optimal two-sample Wald test to detect possible DE genes by estimating model parameters and calculating the P-value. In this paper, we used *ROSeq* function of ROSeq R package to execute this method.

#### 4.2.8. DEF-scRNA-seq

DEF-scRNA-seq is a new DEA method extended by machine learning algorithm. It is based on information entropy and the basic idea is to reflect gene differential expression levels between cellular groups by calculating overall entropy-like function values. It can mainly be divided into two steps: normalization and calculation of the overall differential entropy-like function.

(1) Normalization

Since we need to calculate the entropy-like function values from the gene expression counts between two groups, it is necessary to eliminate the effect of sequencing depth. Therefore, the count matrix is normalized at the gene level first:

$${\tilde{y}}_{ij}=\frac{y_{ij}}{\sum_{i=1}^{n}y_{ij}}$$ (17)

(2) Calculation of DEF

To simplify the computational model, we consider original read counts of specific genes to be random variables, and the sum of gene read counts in each group can be viewed as the probability of variable occurrence in each group (Eq (18)). Then the entropy-like function of gene *i* can be defined as Eq (19):

$$p_{j}=\frac{{\tilde{y}}_{ij}}{\sum_{i=1}^{n}{\tilde{y}}_{ij}}$$ (18)

$$Ent(Y)=1-\frac{-\sum_{j=1}^{n}p_{j}\log p_{j}}{\log n}$$ (19)

From Eq (19) we can see that if gene *i* is expressed with the same level across all samples, that is ${\tilde{y}}_{i1}={\tilde{y}}_{i2}=\cdots ={\tilde{y}}_{in}$, then:

$$Ent(Y)=1-\frac{-\sum_{j=1}^{n}\frac{1}{n}\log\frac{1}{n}}{\log n}=0$$ (20)

which shows this gene is not differentially expressed, giving practically coincident results with the fact that gene *i* have the same expression level across all samples. In contrast, a higher DEF value indicates a higher level of gene differential expression, and such genes should be considered as DE genes if DEF value is larger than a reasonable threshold. Thus, we can detect potential DE genes and the results are reliable.

In this paper, we conducted our own differential entropy-like function in R software based on the above theory.

### 4.3. Evaluation metrics

For the expression of each gene in the gene-cell expression matrix, the following hypothesis test can be proposed:

*H*_0_: *μ*_1_ = *μ*_2_, which means this gene is non-DE gene.
*H*_1_: *μ*_1_ ≠ *μ*_2_, which means this gene is DE gene.

For algorithms with nominal P-values such as DESeq2 and Limma, genes satisfying P-value < 0.05 were selected as predicted DE genes at the significance level α = 0.05; The DEF method based on information entropy cannot directly obtain the P-value of the test, we can only calculate the DEF value of each gene based on original read counts. Generally, the larger DEF function value is, the more likely it may be DE genes, so the top P genes with the largest DEF value were selected as predicted DE genes in this paper. For simulated datasets, P = 1000; while in real scRNA-seq datasets, we can choose the value of P according to our expected demands. Finally, we can obtain the confusion matrix shown in Table 4.

**Table 4:** Confusion matrix of different methods.

Predict True	Not reject *H*_0_	Reject *H*_0_	Total
*H*_0_ is True	TN	FP	N
*H*_0_ is False	FN	TP	P
Total	N*	P*	

In the evaluation of DEA methods, we not only want to uncover as many potentially DE genes as possible, but more focused on the proportion of true positive and false positive genes among the predicted DE genes, as well as the reliability of the algorithm when determining DE genes. Therefore, we used the following indicators to evaluate the efficacy of different DEA methods:

1. True positive rate, which represents the ability of the algorithm to correctly identify DE genes.
2. False positive rate (Type I error rate), which represents the proportion of DE genes incorrectly identified by the algorithm.
3. F1-score, which is harmonic mean of precision and recall, and can objectively reflect the overall performance of the classification algorithm.
4. Mean-Rank, calculated as the rank sum of the true DE genes divided by the number of predicted differential genes P*.
5. Mean Rank-sum Ratio (MRSR). Since the absolute value of Mean-Rank is affected by TP value and is not comparable in different cases, so we weight the Mean-Rank and obtain the dimensionless statistic MRSR on [1, +∞]. A smaller MRSR means the higher reliability of the algorithm when detecting DE genes.
6. ROC curve and AUC values.

The performance metrics mentioned above were computed by comparing the predicted DE genes of each DEA method with referencing genes generated through simulation process using Eqs (21) ~ (24):

$$TPR=\frac{TP}{TP+FN}$$ (21)

$$FPR=\frac{FP}{FP+TN}$$ (22)

$$F1-score=\frac{2\times precision\times recall}{precision+recall}=\frac{2TP}{2TP+FP+FN}$$ (23)

$$MRSR=\frac{Mean-Rank}{(TP+1)/2}$$ (24)

## 5. Results and discussion

### 5.1. Test on simulated datasets

**Fig 6:**
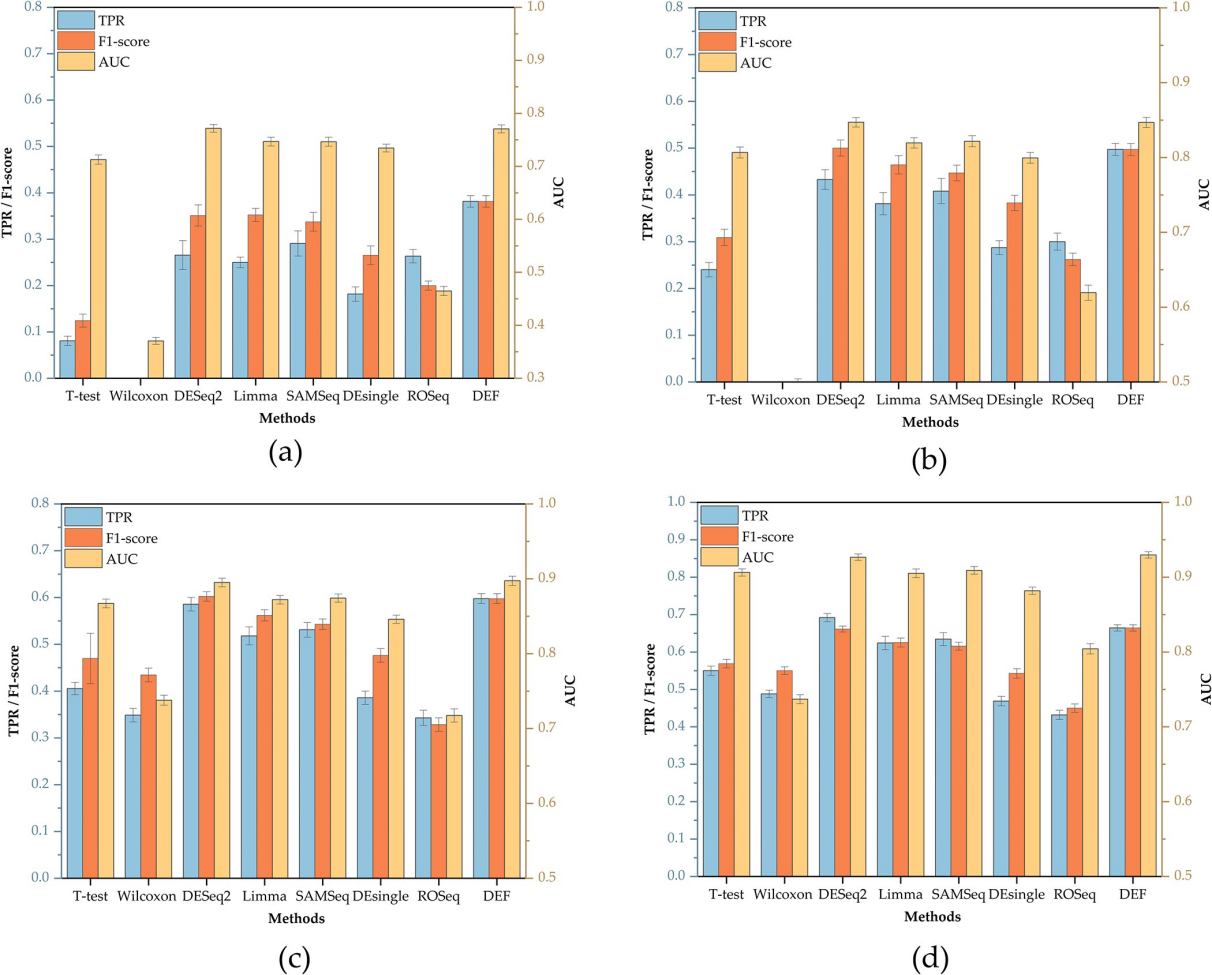
Positive evaluation indicators of each DEA method on simulated datasets under different biological replications (K). (a) K = 2. (b) K = 3. (c) K = 4. (d) K = 5.

**Fig 7:**
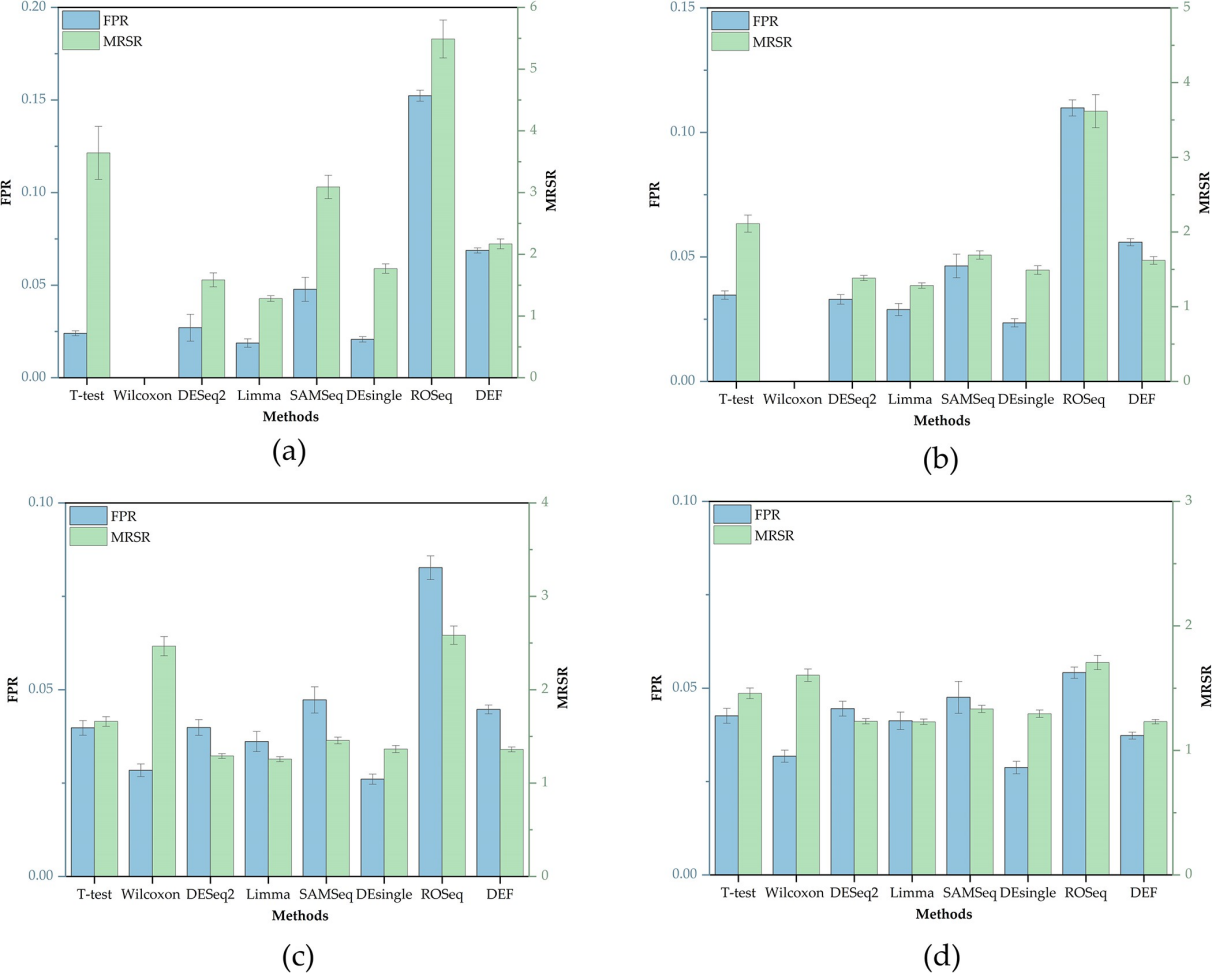
Negative evaluation indicators of each DEA method on simulated datasets under different biological replications (K). (a) K = 2. (b) K = 3. (c) K = 4. (d) K = 5.

**Fig 8:**
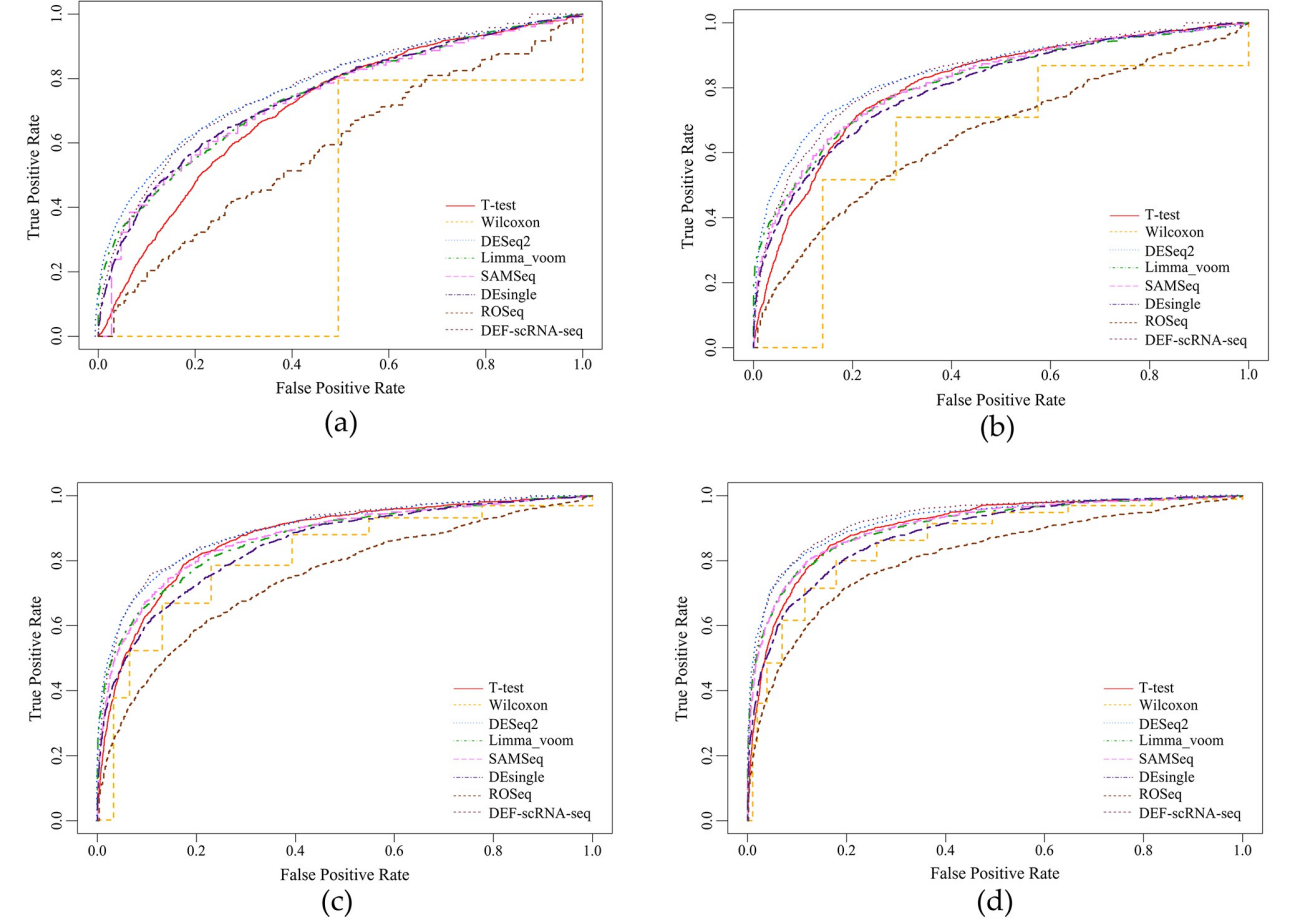
ROC curves of each DEA method on simulated datasets under different biological replications (K). (a) K = 2. (b) K = 3. (c) K = 4. (d) K = 5.

For each evaluation metric, 30 different sets of values can be obtained according to the previously designed experimental scheme, the original result can be found in S1 Table. In this paper, we focused on the algorithm’s accuracy as well as stability, so we calculated the mean and standard deviation of 30 values obtained in each simulation separately. Fig 6 shows TPR, F1-score, and AUC values of each algorithm under different biological replications, which are all positive evaluation indicators; Fig 7 shows FPR and MRSR values of each algorithm under different biological replications, which are all negative evaluation indicators; Fig 8 shows the ROC curves of each algorithm.

The simulation results suggested that general DEA methods are weak in detecting DE genes. For example, Wilcoxon rank sum test tends to predict all genes as non-DE genes under small biological replicate conditions, which obviously cannot meet the needs of practical applications. The performance of the DEA methods which are extended from bulk RNA-seq is equal, both parametric DEA methods like DESeq2, Limma_voom and the non-parametric method like SAMSeq can achieve ideal results. These methods can produce relatively high TPR values as well as low FPR values, and their overall AUC values are almost greater than 0.8, indicating a strong ability to detect potential DE genes. Among new DEA methods exclusively developed for scRNA-seq, the performance varies greatly. ROSeq, for example, has a higher FPR value which is always greater than 0.05, and MRSR value of this approach is also higher compared to other DEA methods, indicating that the probability of misclassification of this method is high and that this method is not suitable for promotion in small biological replicate conditions. In contrast, the DEF-scRNA-seq algorithm based on information entropy has the highest TPR and AUC values among all these methods when K<4; As the number of biological replicates increases, the advantage of this algorithm is slightly diminished, it tends to be as effective as the classic DESeq2 algorithm, whose TPR value may even slightly lower than the classic DESeq2 algorithm, but it still beats other conventional techniques. Additionally, its FPR is also consistently less than 0.05, demonstrating clear advantages over traditional DEA methods.

Since the number of DEA methods and evaluation metrics involved in this paper are large, it is difficult to find an algorithm that makes each evaluation index value optimal. In order to compare these algorithms objectively, the entropy weight method (EWM) was chosen to weight these values and finally obtain the comprehensive scores of each algorithm under different conditions, as shown in Table 5 From Table 5 we can find that the comprehensive scores of DEseq2, Limma_voom, SAMSeq and other DEA methods extended from bulk RNA-seq are relatively high, indicating that the overall performance of these algorithms are superior; while the comprehensive scores of DEF-scRNA-seq algorithm based on information entropy are always in top two under different biological replicate conditions, indicating that the algorithm is more adaptable to small biological replications, and such algorithm is worthy of further development.

**Table 5:** Comprehensive score of each DEA methods under different biological replications (K).

DEA Methods	K = 2	K = 3	K = 4	K = 5
DEF-scRNA-seq	**92.6**	93.6	87.5	**92.2**
DESeq2	89.0	**94.9**	**91.5**	87.1
Limma_voom	88.1	89.5	87.5	79.9
SAMSeq	82.1	85.6	76.6	70.6
DEsingle	75.6	78.7	80.1	67.1
T-test	53.1	68.2	69.8	59.5
ROSeq	36.4	37.6	0	8.6
Wilcoxon	0	0	45.0	34.9

### 5.2. Test on real scRNA-seq dataset

Based on the above simulation analysis results, we obtained the overall performance of each DEA method under different biological replications. To further examine whether these methods also achieve the desired results in real scenario, we therefore conducted an empirical analysis using real scRNA-seq dataset with the help of the publicly available datasets.

**Fig 9:**
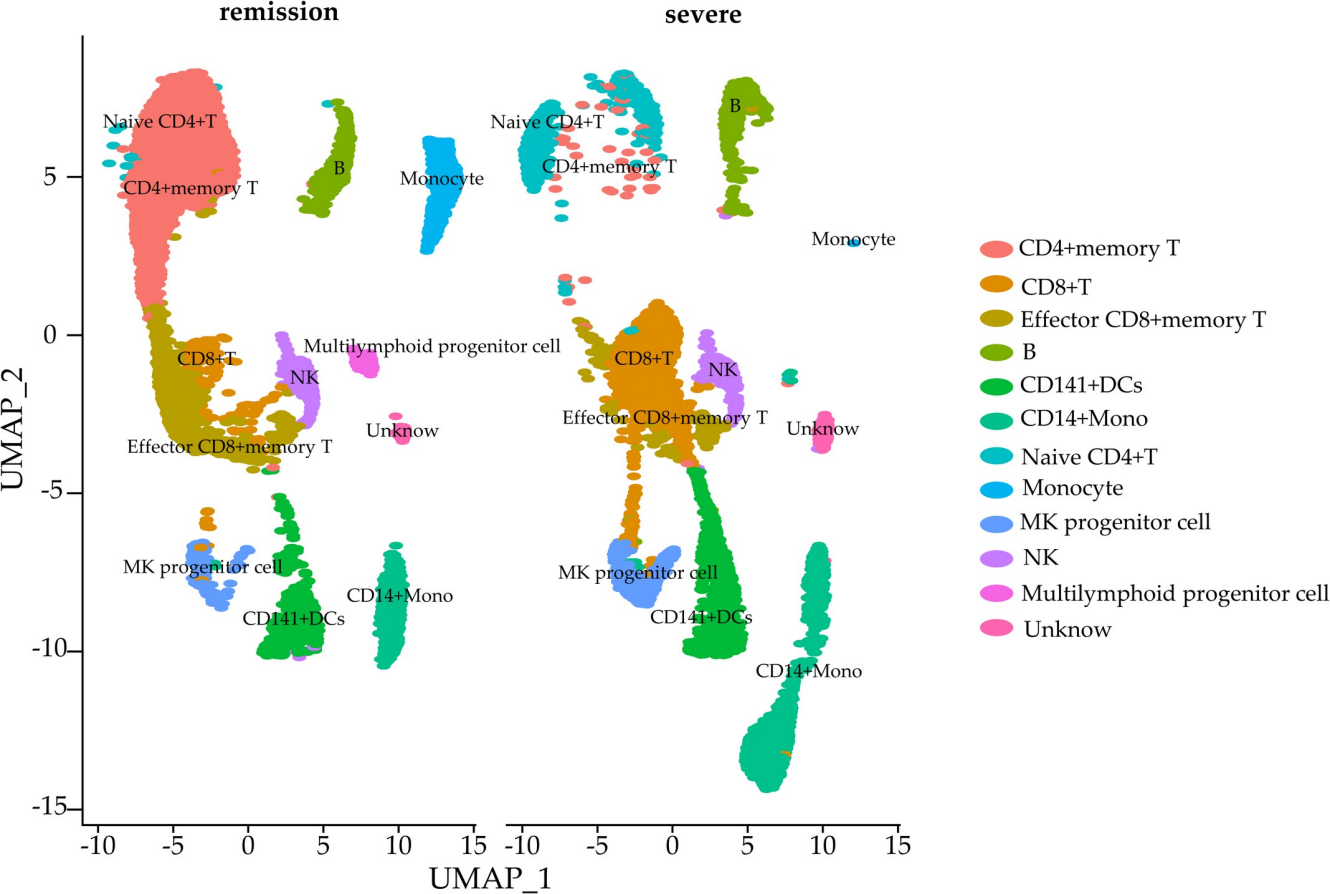
Spectral clustering results of PBMCs.

Take the dataset GSE150861 as example, in order to examine whether PBMCs in our research changes significantly before and after treatment in morphology, we first categorized all cells and explored their subtypes. As mentioned above, we used spectral clustering to assign cells into clusters, which is a type of graph-based clustering method that is advantageous in clustering sparse data. As shown in Fig 9, we finally divided all cells into 12 clusters and annotated their subgroups with the help of CellMarker database. Obviously, there were inconsistencies in the types and numbers of subpopulations caused by differential gene expression in PBMCs of COVID-19 patients in severe and remission. For example, the number of Memory CD4+ T cells and Effector CD8+ T cells in PBMCs of patients in remission increased substantially, which indicated that Tocilizumab treatment induced an immune cell antiviral response, resulting in overactive expression of relevant genes. Therefore, analysis of above data to detect DE genes across different cellular groups was important for assessing drug efficacy and laying the groundwork for clinical treatment of this disease.

**Fig 10:**
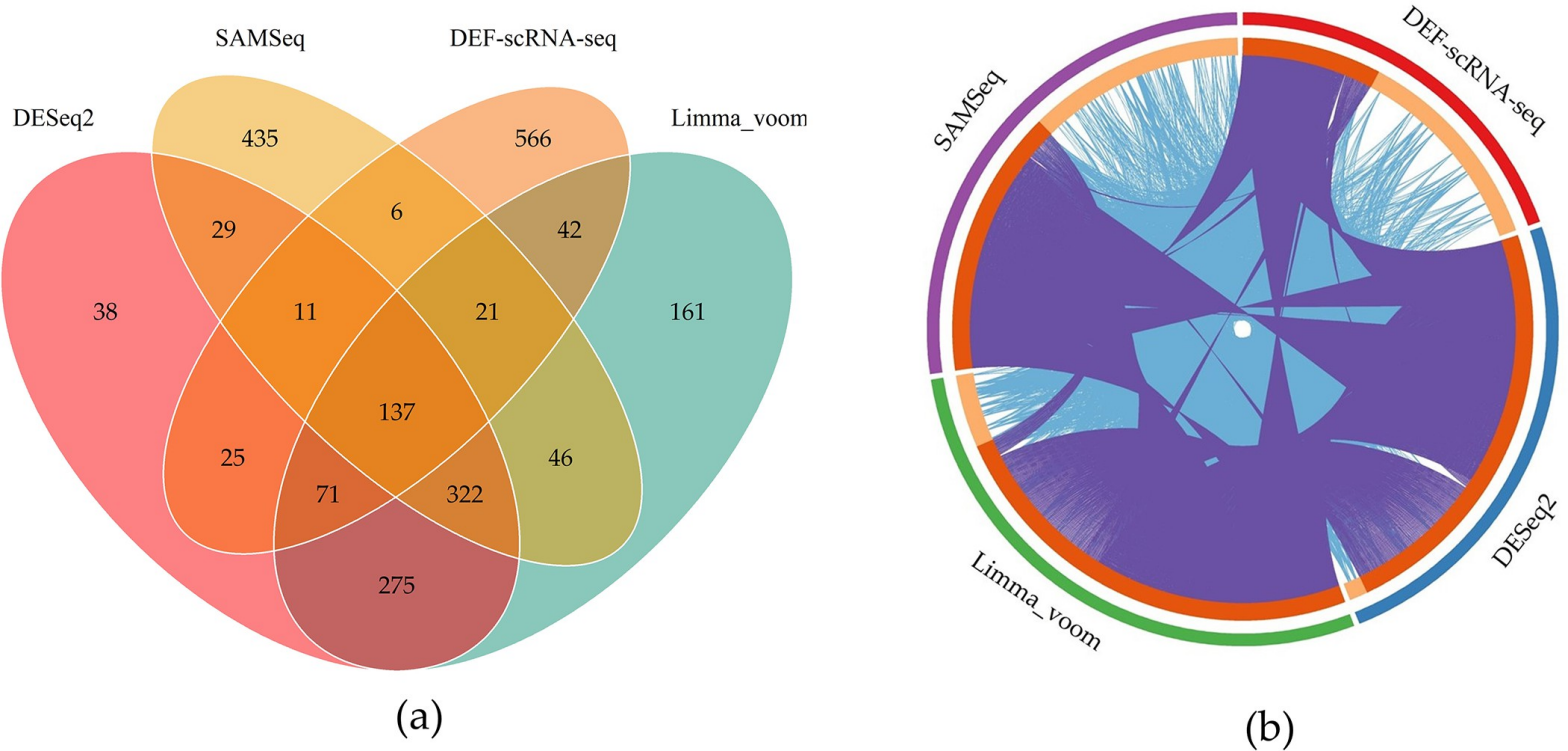
DE genes detected by different DEA algorithms. (a) Venn diagram. (b) Circos plot.

Due to the biological replication number in this dataset was three, only DESeq2, Limma_voom, SAMSeq and DEF-scRNA-seq algorithms were considered for the comparison of different DEA methods on this dataset based on the results of simulated experiments. Finally, DESeq2 detected 908 DE genes including *SMIM1*, etc.; Limma_voom detected 1075 DE genes including *TNFAIP3*, etc.; SAMSeq detected 1007 DE genes including *SDF4*, etc.; and DEF-scRNA-seq algorithm detected 879 DE genes including *CAMP*, etc. Fig 10(A) depicts a Venn diagram based on the above results, which present the overlap of DE genes discovered by different DEA methods. We note that DE genes discovered by DESeq2 were to a large extent found also by Limma, which share 805 common genes and only find 38 and 161 unique genes that were not shared with other methods, while SAMSeq and DEF-scRNA-seq find a large number of unique genes up to 435 and 566. only 137 genes are judged as DE genes by all these algorithms, by using these overlapping genes for subsequent research, we may obtain a more representative result. To further examine whether DE genes detected by different DEA methods have similar functions, we conduct Gene Set Enrichment Analysis, which can link gene function with its phenotype. The Circos plot (Fig 10(B)) illustrates how DE genes detected by different DEA algorithms overlap. Purple lines link the same gene that is shared by multiple gene sets, whereas blue lines link different genes that fall into the same Gene Ontology (GO) term in enrichment analysis, indicating they may have similar functions. The Circos plot indicated that though multiple DEA methods share a large number of DE genes with the same function, there exists certain unique DE genes or GO terms that can only be found by particular methods. Thus, whether the DEA method used is reasonable or not has a significant impact on the accuracy of the results.

Since the true DE genes were not given in this dataset, in order to evaluate whether the above methods can also achieve the expected results in real scenario, the initial definition of DE genes was used to obtain the reference genes. Here, we used Fold Change as evaluation criteria and genes satisfying |log2*FoldChange*|>1 were selected as the reference genes [53]. Finally, 839 reference genes were screened in total. Accordingly, we evaluated the above four algorithms based on the reference genes screened and the results were shown in Table 6 In addition to TPR, FPR and F1-score, we also considered the FDR value of these methods in real datasets, which was calculated by *FP*/(*FP*+*TP*) and means the proportion of false discoveries in predicted DE genes. From Table 6 we can find the above results: Firstly, it seems that all these methods have a relatively higher FDR value, which is consistent with Li’s work where they indicated that the median estimated FDR of current DEA approaches ranges from 0.25 to 0.7 when biological replicate is limited to three in each group [54]. Just as Squair et al. (2021) mentioned in the literature, false discovery is a universal phenomenon that may compromise the biological interpretation of a single-cell experiment and elucidate the progression of mechanisms that may cause the above issue [55]. Secondly, many existing studies have revealed the advantages of bulk sequencing methods. For instance, Wang et al. (2019) indicated that current methods designed for scRNA-seq data do not show better performance than bulk RNA-seq methods [56]; Heumos et al. (2023) suggest single-cell specific methods may underestimate the variance of gene expression and may wrongly identify high-expressed genes as DE genes. Thus, they recommend using flexible methods like DESeq2 or Limma under complex experiment designs [57]. During our simulation and real study, we agree that bulk RNA-seq methods are still competitive. However, we also find that DEF-scRNA-seq method which was extended by machine learning algorithms performs better among all these methods, with a higher F1-score value and lower FDR value. We speculate that this may because DEF-scRNA-seq has the ability to handle low expression counts, especially zero counts, which is of vital importance to satisfying main characteristics of scRNA-seq data, making it possible to explore the true DE genes while reduce FPR and FDR as much as possible. Therefore, we believe such methods have a greater application value in the future development of this field.

**Table 6:** Evaluation of different DEA methods on two real scRNA-seq datasets.

Datasets	Methods	TPR	FPR	FDR	F1-score
GSE150861	DESeq2	0.359	0.019	0.669	0.345
	Limma_voom	0.445	0.021	0.653	0.390
	SAMSeq	0.285	0.023	0.763	0.259
	DEF-scRNA-seq	**0.547**	**0.013**	**0.478**	**0.534**
GSE235436	DESeq2	0.374	0.064	0.594	0.389
	Limma_voom	**0.790**	0.070	0.425	**0.666**
	SAMSeq	0.350	**0.033**	0.441	0.430
	DEF-scRNA-seq	0.654	0.043	**0.346**	**0.666**

Single-cell study is one of the most noteworthy technological fields in the recent years. With the development of high-throughput sequencing technology, numerous sequencing data and various analytical methods are available for bioinformaticians. We use well-established pipelines to evaluate performance of various DEA methods and our results demonstrate that different pipelines have varying effects based on the tools used throughout the pipeline as well as input data, which supports previous studies [58]. Similarly, Knight et al. (2023) developed Integrated Benchmarking scRNA-seq Analytical Pipeline (IBRAP), which can also help determining the optimal pipeline combinations for their data [59]. With the help of these pipelines, we can find the best matching analytical method for certain data.

## 6.Conclusions

In this paper, we classified existing DEA methods into three major categories and seven subcategories based on their original data motivation, distributional assumptions, and statistical models, etc. On this basis, we evaluated and compared eight different DEA methods and mainly focused on their performance under small biological replicate conditions. The main conclusions are as follows. First, among the above methods, no single method is optimal under all conditions, and different results can be obtained even with the same biological replicates if different evaluation metrics are used, therefore the choice of DEA methods in a specific situation is dependent on the real experimental conditions. Second, DEA methods extended by bulk RNA-seq like DESeq2, Limma_voom and SAMSeq remain competitive under small biological replicate conditions, which can obtain relatively higher TPR and AUC values while controlling FPR at a lower level. Third, the newly developed DEA method DEF-scRNA-seq based on information entropy consistently ranks first and second among all the above algorithms in terms of comprehensive scores under all conditions. Thus, such algorithm has great potential for further promotion.

Single-cell sequencing is an emerging field in genomics and plays a significant role in medical fields such as immunology, oncology, and neurology, among others. As a key component of sequencing data analysis, DEA can identify gene expression patterns from massive data, which is important for revealing cellular heterogeneity problems or diseases pathogenesis. With the development of sequencing technology, the dimensionality of expression matrix is becoming larger and larger, and at the same time single-cell detection technologies incorporating multiple histology are gradually being applied, making traditional DEA methods unable to fulfill the demands of analysis at the new stage. From the results of this paper, we can reasonably speculate the application value of machine learning algorithms in this field, so the next stage of research can focus on transcriptome analysis combined with machine learning algorithms, which is also the development trend of this field in the era of big data.

## Supporting information

S1 Table Simulation results of DEA methods under different biological replicates. (PDF)

S1 File Code script of our experiments. (R)
