# Supplementary material for: Comparative study on differential expression analysis methods for single-cell RNA sequencing data with small biological replicates: Based on single-cell transcriptional data of PBMCs from COVID-19 severe patients
Source: PLoS One. 2024 Mar 27;19(3):e0299358. doi: 10.1371/journal.pone.0299358 (PMC10971542; doi:10.1371/journal.pone.0299358)
Supplement: S1 Table — (PDF) [file pone.0299358.s001.pdf]

Table S1.1 Simulation results of T-test under different biological replicates (K).

			T-test			
K	Round	TP	FP	F1-score	MRSR	AUC
2	1	73	215	0.113	3.81	0.699
	2	89	218	0.136	3.51	0.705
	3	60	208	0.095	4.26	0.697
	4	79	226	0.121	3.95	0.712
	5	90	221	0.137	3.45	0.715
	6	75	217	0.116	3.53	0.706
	7	88	215	0.135	3.55	0.717
	8	92	196	0.143	3.33	0.725
	9	82	219	0.126	3.37	0.722
	10	82	203	0.128	3.47	0.711
	11	74	210	0.115	3.65	0.720
	12	75	211	0.117	3.63	0.711
	13	88	247	0.132	3.73	0.729
	14	67	228	0.103	4.85	0.710
	15	74	214	0.115	3.76	0.717
	16	83	215	0.128	3.79	0.707
	17	63	223	0.098	4.5	0.701
	18	77	201	0.121	3.59	0.709
	19	70	216	0.109	4.42	0.708
	20	82	230	0.125	3.81	0.718
	21	74	217	0.115	3.65	0.698
	22	93	235	0.140	3.38	0.709
	23	85	208	0.131	3.23	0.717
	24	83	233	0.126	3.29	0.720
	25	78	213	0.121	3.62	0.709
	26	104	211	0.158	2.82	0.731
	27	85	200	0.132	3.3	0.711
	28	72	197	0.113	3.75	0.724
	29	92	228	0.139	3.14	0.708
	30	93	205	0.143	3.13	0.714
3	1	216	319	0.281	2.10	0.807
	2	245	297	0.318	2.11	0.800
	3	224	319	0.290	2.28	0.800
	4	238	327	0.304	2.18	0.810
	5	258	317	0.328	2.05	0.813
	6	230	295	0.302	1.95	0.800
	7	237	302	0.308	2.09	0.808
	8	253	307	0.324	2.10	0.805
	9	221	299	0.291	2.24	0.794
	10	243	320	0.311	2.16	0.807
	11	233	321	0.300	2.21	0.812
	12	219	309	0.287	2.20	0.789
	13	278	326	0.347	2.03	0.816
	14	242	313	0.311	2.17	0.817
	15	259	273	0.338	1.94	0.810
	16	233	323	0.299	2.17	0.814
	17	266	299	0.340	1.97	0.805

	18	223	323	0.288	2.33	0.803
	19	255	288	0.331	1.91	0.814
	20	248	333	0.314	2.12	0.806
	21	243	321	0.311	2.06	0.797
	22	214	326	0.278	2.27	0.797
	23	243	294	0.316	2.11	0.808
	24	237	326	0.303	2.07	0.818
	25	234	327	0.300	2.26	0.803
	26	244	339	0.308	2.15	0.808
	27	228	297	0.299	2.13	0.802
	28	260	303	0.333	1.84	0.820
	29	233	304	0.303	2.04	0.809
	30	240	311	0.309	2.12	0.806
4	1	388	350	0.446	1.71	0.863
	2	430	364	0.479	1.66	0.869
	3	420	375	0.468	1.70	0.864
	4	402	353	0.458	1.64	0.864
	5	408	364	0.460	1.68	0.870
	6	402	310	0.470	1.57	0.872
	7	407	380	0.456	1.70	0.865
	8	386	358	0.443	1.62	0.856
	9	434	380	0.479	1.68	0.861
	10	400	346	0.458	1.62	0.870
	11	411	364	0.463	1.68	0.875
	12	387	379	0.438	1.73	0.865
	13	414	385	0.460	1.69	0.859
	14	404	346	0.462	1.63	0.867
	15	398	332	0.460	1.60	0.868
	16	430	358	0.481	1.66	0.875
	17	417	360	0.469	1.61	0.867
	18	404	338	0.464	1.63	0.879
	19	407	359	0.461	1.63	0.869
	20	402	367	0.454	1.68	0.864
	21	406	374	0.456	1.69	0.872
	22	397	358	0.452	1.69	0.866
	23	413	327	0.475	1.54	0.869
	24	416	337	0.475	1.58	0.870
	25	383	372	0.436	1.76	0.854
	26	414	365	0.465	1.72	0.874
	27	386	380	0.437	1.74	0.874
	28	391	358	0.447	1.65	0.858
	29	400	342	0.459	1.65	0.866
	30	405	358	0.459	1.65	0.867
5	1	556	393	0.571	1.49	0.906
	2	555	395	0.569	1.44	0.907
	3	552	402	0.565	1.52	0.902
	4	553	396	0.567	1.46	0.908
	5	547	388	0.565	1.53	0.903
	6	555	375	0.575	1.44	0.909
	7	559	369	0.580	1.43	0.907

	8	550	391	0.567	1.47	0.910
	9	544	364	0.570	1.42	0.906
	10	552	387	0.569	1.42	0.906
	11	574	391	0.584	1.44	0.910
	12	529	381	0.554	1.47	0.905
	13	564	367	0.584	1.44	0.912
	14	561	359	0.584	1.41	0.912
	15	549	349	0.579	1.42	0.913
	16	554	379	0.573	1.44	0.903
	17	541	373	0.565	1.43	0.906
	18	540	377	0.563	1.46	0.906
	19	573	375	0.588	1.45	0.917
	20	530	357	0.562	1.47	0.906
	21	538	424	0.548	1.58	0.910
	22	533	414	0.548	1.52	0.899
	23	532	380	0.556	1.49	0.897
	24	537	413	0.551	1.52	0.901
	25	536	387	0.557	1.42	0.898
	26	564	377	0.581	1.43	0.912
	27	566	372	0.584	1.46	0.908
	28	543	396	0.560	1.40	0.898
	29	560	406	0.570	1.48	0.906
	30	551	362	0.576	1.40	0.906

Table S1.2 Simulation results of Wilcoxon under different biological replicates (K).

			Wilcoxon			
K	Round	TP	FP	F1-score	MRSR	AUC
	1	0	0	0	NA	0.365
	2	0	0	0	NA	0.360
	3	0	0	0	NA	0.360
	4	0	0	0	NA	0.362
	5	0	0	0	NA	0.383
	6	0	0	0	NA	0.359
	7	0	0	0	NA	0.372
	8	0	0	0	NA	0.376
	9	0	0	0	NA	0.372
	10	0	0	0	NA	0.368
2	11	0	0	0	NA	0.376
	12	0	0	0	NA	0.368
	13	0	0	0	NA	0.387
	14	0	0	0	NA	0.373
	15	0	0	0	NA	0.373
	16	0	0	0	NA	0.366
	17	0	0	0	NA	0.372
	18	0	0	0	NA	0.363
	19	0	0	0	NA	0.366
	20	0	0	0	NA	0.372
	21	0	0	0	NA	0.365

	22	0	0	0	NA	0.367
	23	0	0	0	NA	0.375
	24	0	0	0	NA	0.376
	25	0	0	0	NA	0.372
	26	0	0	0	NA	0.377
	27	0	0	0	NA	0.373
	28	0	0	0	NA	0.378
	29	0	0	0	NA	0.371
	30	0	0	0	NA	0.368
3	1	0	0	0	NA	0.508
	2	0	0	0	NA	0.487
	3	0	0	0	NA	0.492
	4	0	0	0	NA	0.493
	5	0	0	0	NA	0.498
	6	0	0	0	NA	0.489
	7	0	0	0	NA	0.498
	8	0	0	0	NA	0.489
	9	0	0	0	NA	0.492
	10	0	0	0	NA	0.494
	11	0	0	0	NA	0.501
	12	0	0	0	NA	0.481
	13	0	0	0	NA	0.515
	14	0	0	0	NA	0.505
	15	0	0	0	NA	0.510
	16	0	0	0	NA	0.498
	17	0	0	0	NA	0.479
	18	0	0	0	NA	0.488
	19	0	0	0	NA	0.501
	20	0	0	0	NA	0.496
	21	0	0	0	NA	0.494
	22	0	0	0	NA	0.487
	23	0	0	0	NA	0.497
	24	0	0	0	NA	0.500
	25	0	0	0	NA	0.489
	26	0	0	0	NA	0.503
	27	0	0	0	NA	0.487
	28	0	0	0	NA	0.506
	29	0	0	0	NA	0.494
	30	0	0	0	NA	0.500
4	1	347	267	0.430	2.53	0.734
	2	373	261	0.457	2.39	0.739
	3	347	274	0.428	2.57	0.730
	4	336	237	0.427	2.41	0.738
	5	333	270	0.415	2.62	0.737
	6	351	248	0.439	2.41	0.739
	7	350	277	0.430	2.57	0.737
	8	360	260	0.444	2.44	0.718
	9	356	266	0.439	2.49	0.737
	10	374	252	0.460	2.34	0.738
	11	368	259	0.452	2.41	0.748

	12	341	258	0.427	2.51	0.736
	13	329	273	0.411	2.65	0.735
	14	360	232	0.452	2.29	0.742
	15	319	238	0.410	2.49	0.736
	16	361	262	0.445	2.45	0.751
	17	360	241	0.450	2.34	0.729
	18	353	248	0.441	2.40	0.750
	19	356	260	0.441	2.46	0.737
	20	358	261	0.442	2.45	0.742
	21	356	275	0.437	2.54	0.742
	22	332	256	0.418	2.54	0.736
	23	353	225	0.447	2.27	0.739
	24	353	233	0.445	2.32	0.743
	25	314	259	0.399	2.65	0.729
	26	344	260	0.429	2.51	0.740
	27	341	261	0.426	2.53	0.745
	28	341	259	0.426	2.51	0.731
	29	334	223	0.429	2.33	0.738
	30	356	278	0.436	2.56	0.737
5	1	481	297	0.541	1.63	0.738
	2	498	295	0.555	1.60	0.737
	3	479	310	0.535	1.70	0.738
	4	498	294	0.556	1.60	0.736
	5	486	299	0.545	1.68	0.734
	6	494	279	0.557	1.59	0.742
	7	496	280	0.559	1.57	0.734
	8	484	265	0.553	1.55	0.744
	9	482	255	0.555	1.49	0.742
	10	481	300	0.540	1.63	0.736
	11	496	283	0.558	1.59	0.747
	12	479	289	0.542	1.61	0.729
	13	508	288	0.566	1.60	0.737
	14	502	276	0.565	1.54	0.744
	15	487	280	0.551	1.60	0.742
	16	493	280	0.556	1.55	0.738
	17	480	277	0.546	1.60	0.737
	18	475	281	0.541	1.59	0.735
	19	492	279	0.556	1.62	0.747
	20	474	268	0.544	1.61	0.733
	21	483	309	0.539	1.68	0.748
	22	476	304	0.535	1.66	0.728
	23	477	285	0.541	1.67	0.728
	24	473	305	0.532	1.64	0.735
	25	484	277	0.550	1.62	0.732
	26	504	285	0.563	1.58	0.736
	27	489	292	0.549	1.66	0.740
	28	496	300	0.552	1.57	0.723
	29	486	296	0.545	1.59	0.734
	30	506	253	0.575	1.50	0.734

Table S1.3 Simulation results of Limma under different biological replicates (K).

Limma						
K	Round	TP	FP	F1-score	MRSR	AUC
2	1	244	167	0.346	1.31	0.742
	2	259	156	0.366	1.25	0.754
	3	220	167	0.317	1.37	0.735
	4	245	178	0.344	1.32	0.747
	5	243	168	0.344	1.23	0.749
	6	248	162	0.352	1.26	0.741
	7	237	172	0.336	1.36	0.753
	8	260	171	0.363	1.26	0.754
	9	242	166	0.344	1.27	0.743
	10	248	165	0.351	1.26	0.751
	11	281	156	0.391	1.30	0.755
	12	254	160	0.359	1.25	0.744
	13	251	230	0.339	1.40	0.765
	14	251	203	0.345	1.37	0.744
	15	258	188	0.357	1.32	0.745
	16	246	138	0.355	1.28	0.734
	17	241	151	0.346	1.31	0.731
	18	254	159	0.360	1.21	0.746
	19	245	157	0.350	1.28	0.741
	20	253	179	0.353	1.25	0.745
	21	248	157	0.353	1.25	0.734
	22	242	186	0.339	1.28	0.745
	23	259	181	0.360	1.26	0.751
	24	228	156	0.329	1.27	0.750
	25	256	188	0.355	1.28	0.746
	26	263	149	0.373	1.24	0.764
	27	263	198	0.360	1.27	0.734
	28	248	123	0.362	1.23	0.754
	29	261	151	0.370	1.24	0.749
	30	249	172	0.350	1.27	0.752
3	1	384	228	0.476	1.26	0.826
	2	391	248	0.477	1.28	0.817
	3	361	245	0.450	1.29	0.817
	4	389	262	0.471	1.30	0.820
	5	373	259	0.457	1.22	0.818
	6	338	248	0.426	1.34	0.807
	7	419	263	0.498	1.27	0.821
	8	370	231	0.462	1.22	0.819
	9	361	259	0.446	1.33	0.810
	10	400	260	0.482	1.29	0.823
	11	410	270	0.488	1.30	0.827
	12	364	299	0.438	1.37	0.807
	13	437	309	0.501	1.33	0.833
	14	353	249	0.441	1.27	0.827
	15	407	311	0.474	1.30	0.824
	16	367	250	0.454	1.25	0.824
	17	380	244	0.468	1.28	0.810

	18	371	244	0.459	1.28	0.813
	19	403	302	0.473	1.34	0.822
	20	375	255	0.460	1.25	0.821
	21	376	277	0.455	1.28	0.810
	22	372	250	0.459	1.25	0.810
	23	358	249	0.446	1.29	0.821
	24	369	250	0.456	1.28	0.830
	25	375	245	0.463	1.29	0.815
	26	421	247	0.505	1.25	0.822
	27	337	233	0.429	1.22	0.815
	28	408	267	0.487	1.27	0.827
	29	386	278	0.464	1.30	0.826
	30	380	250	0.466	1.27	0.821
4	1	551	356	0.578	1.27	0.875
	2	534	353	0.566	1.25	0.874
	3	520	321	0.565	1.22	0.871
	4	488	288	0.550	1.20	0.866
	5	540	332	0.577	1.26	0.876
	6	497	276	0.561	1.24	0.871
	7	524	325	0.567	1.24	0.871
	8	479	294	0.540	1.25	0.860
	9	518	370	0.549	1.32	0.866
	10	527	326	0.569	1.24	0.877
	11	509	322	0.556	1.25	0.877
	12	525	302	0.575	1.26	0.870
	13	529	330	0.569	1.27	0.865
	14	527	335	0.566	1.25	0.873
	15	506	332	0.551	1.25	0.874
	16	537	335	0.574	1.28	0.881
	17	512	294	0.567	1.22	0.871
	18	517	288	0.573	1.22	0.883
	19	532	344	0.567	1.25	0.876
	20	481	326	0.532	1.29	0.867
	21	510	323	0.556	1.25	0.879
	22	550	342	0.581	1.27	0.876
	23	506	288	0.564	1.23	0.865
	24	501	303	0.555	1.24	0.869
	25	490	330	0.538	1.29	0.860
	26	546	361	0.573	1.32	0.876
	27	527	342	0.564	1.27	0.879
	28	514	325	0.559	1.25	0.869
	29	508	327	0.554	1.26	0.872
	30	531	360	0.562	1.27	0.871
5	1	629	416	0.615	1.25	0.908
	2	618	355	0.626	1.22	0.904
	3	623	347	0.632	1.21	0.902
	4	627	378	0.625	1.25	0.902
	5	607	368	0.615	1.23	0.903
	6	629	340	0.639	1.23	0.906
	7	629	368	0.630	1.20	0.909

	8	636	346	0.642	1.21	0.912
	9	592	349	0.610	1.20	0.905
	10	601	366	0.611	1.26	0.900
	11	664	388	0.647	1.23	0.911
	12	613	364	0.620	1.23	0.904
	13	615	323	0.635	1.19	0.905
	14	632	358	0.635	1.20	0.910
	15	653	363	0.648	1.20	0.914
	16	606	405	0.603	1.27	0.901
	17	626	389	0.621	1.24	0.908
	18	618	383	0.618	1.22	0.902
	19	648	412	0.629	1.24	0.914
	20	599	368	0.609	1.25	0.902
	21	633	385	0.627	1.25	0.914
	22	616	386	0.615	1.24	0.899
	23	617	364	0.623	1.25	0.894
	24	597	369	0.607	1.27	0.902
	25	617	375	0.619	1.23	0.894
	26	658	391	0.642	1.22	0.916
	27	647	387	0.636	1.24	0.909
	28	611	354	0.622	1.21	0.897
	29	627	364	0.630	1.21	0.904
	30	632	373	0.630	1.23	0.904

Table S1.4 Simulation results of DESeq2 under different biological replicates (K).

DESeq2						
K	Round	TP	FP	F1-score	MRSR	AUC
	1	226	156	0.327	1.45	0.771
	2	277	304	0.350	1.72	0.770
	3	271	291	0.347	1.80	0.759
	4	287	285	0.365	1.66	0.768
	5	295	284	0.374	1.59	0.773
	6	268	252	0.353	1.70	0.761
	7	303	321	0.373	1.68	0.780
	8	295	278	0.375	1.58	0.780
	9	220	163	0.318	1.48	0.775
	10	303	298	0.379	1.66	0.776
2	11	300	254	0.386	1.58	0.776
	12	216	141	0.318	1.46	0.778
	13	274	312	0.346	1.68	0.785
	14	226	174	0.323	1.59	0.769
	15	291	295	0.367	1.64	0.770
	16	256	273	0.335	1.75	0.759
	17	273	299	0.347	1.69	0.759
	18	245	155	0.350	1.43	0.771
	19	197	150	0.293	1.45	0.769
	20	228	134	0.335	1.32	0.772
	21	217	147	0.318	1.45	0.763

	22	294	295	0.370	1.61	0.768
	23	280	280	0.359	1.64	0.770
	24	290	300	0.365	1.64	0.774
	25	290	293	0.366	1.60	0.769
	26	265	158	0.372	1.45	0.783
	27	230	169	0.329	1.45	0.762
	28	272	284	0.350	1.64	0.784
	29	286	271	0.367	1.61	0.777
	30	292	276	0.372	1.55	0.775
3	1	429	297	0.497	1.33	0.850
	2	425	261	0.504	1.33	0.847
	3	409	273	0.486	1.38	0.840
	4	440	305	0.504	1.38	0.851
	5	464	328	0.518	1.41	0.853
	6	390	304	0.460	1.45	0.839
	7	471	310	0.529	1.39	0.851
	8	427	273	0.502	1.32	0.847
	9	405	293	0.477	1.44	0.836
	10	439	283	0.510	1.37	0.851
	11	419	295	0.489	1.34	0.854
	12	392	312	0.460	1.45	0.836
	13	459	290	0.525	1.36	0.855
	14	398	292	0.471	1.43	0.850
	15	436	294	0.504	1.38	0.850
	16	454	320	0.512	1.39	0.854
	17	447	291	0.514	1.37	0.840
	18	436	283	0.507	1.36	0.841
	19	438	291	0.507	1.38	0.847
	20	441	313	0.503	1.38	0.850
	21	427	309	0.492	1.39	0.839
	22	437	306	0.501	1.42	0.839
	23	411	260	0.492	1.35	0.849
	24	454	336	0.507	1.42	0.859
	25	418	304	0.485	1.40	0.841
	26	443	279	0.515	1.35	0.844
	27	422	288	0.494	1.39	0.842
	28	458	302	0.520	1.36	0.856
	29	435	304	0.500	1.40	0.853
	30	456	296	0.521	1.36	0.848
4	1	595	373	0.605	1.29	0.897
	2	604	369	0.612	1.29	0.902
	3	571	360	0.591	1.28	0.892
	4	568	351	0.592	1.29	0.892
	5	601	341	0.619	1.26	0.899
	6	575	308	0.611	1.25	0.898
	7	602	378	0.608	1.30	0.898
	8	569	364	0.589	1.32	0.884
	9	586	382	0.596	1.30	0.890
	10	603	363	0.613	1.26	0.901
	11	599	374	0.607	1.32	0.900

	12	587	340	0.609	1.28	0.895
	13	598	357	0.612	1.33	0.884
	14	586	362	0.602	1.30	0.893
	15	565	356	0.588	1.29	0.896
	16	609	372	0.615	1.30	0.901
	17	594	359	0.608	1.25	0.897
	18	585	359	0.602	1.28	0.905
	19	571	365	0.590	1.29	0.895
	20	575	359	0.595	1.30	0.892
	21	603	367	0.612	1.28	0.903
	22	586	336	0.610	1.26	0.895
	23	576	312	0.610	1.24	0.891
	24	587	341	0.609	1.27	0.895
	25	550	361	0.576	1.32	0.882
	26	605	401	0.603	1.34	0.899
	27	587	376	0.598	1.34	0.901
	28	584	354	0.603	1.28	0.887
	29	571	354	0.593	1.29	0.895
	30	577	372	0.592	1.30	0.894
5	1	710	424	0.665	1.27	0.927
	2	698	415	0.661	1.22	0.929
	3	694	401	0.663	1.24	0.919
	4	691	413	0.657	1.23	0.928
	5	687	423	0.651	1.28	0.924
	6	695	390	0.667	1.22	0.928
	7	687	394	0.660	1.22	0.926
	8	688	385	0.664	1.21	0.931
	9	676	370	0.661	1.20	0.927
	10	700	408	0.664	1.24	0.928
	11	710	389	0.677	1.22	0.931
	12	683	392	0.658	1.23	0.927
	13	691	396	0.662	1.22	0.932
	14	692	383	0.667	1.20	0.930
	15	703	373	0.677	1.19	0.934
	16	681	404	0.653	1.23	0.923
	17	675	382	0.656	1.24	0.927
	18	688	412	0.655	1.25	0.925
	19	693	428	0.653	1.25	0.932
	20	705	385	0.675	1.24	0.927
	21	697	419	0.659	1.26	0.934
	22	689	424	0.652	1.24	0.922
	23	665	387	0.648	1.24	0.916
	24	689	396	0.661	1.25	0.925
	25	682	392	0.658	1.24	0.919
	26	702	383	0.673	1.22	0.929
	27	711	426	0.665	1.24	0.927
	28	681	424	0.647	1.26	0.921
	29	705	422	0.663	1.24	0.928
	30	690	377	0.668	1.22	0.925

Table S1.5 Simulation results of SAMSeq under different biological replicates (K).

SAMSeq						
K	Round	TP	FP	F1-score	MRSR	AUC
2	1	306	465	0.346	3.13	0.738
	2	294	447	0.338	3.11	0.741
	3	265	468	0.306	3.52	0.729
	4	294	484	0.331	3.13	0.749
	5	309	433	0.355	2.96	0.751
	6	293	431	0.340	3.02	0.740
	7	304	457	0.345	3.00	0.754
	8	310	451	0.352	2.93	0.753
	9	292	449	0.335	3.13	0.750
	10	323	424	0.370	2.81	0.748
	11	324	414	0.373	2.85	0.752
	12	205	245	0.283	3.38	0.748
	13	226	303	0.296	3.67	0.764
	14	302	450	0.345	3.10	0.747
	15	313	499	0.345	3.19	0.748
	16	291	455	0.333	3.15	0.738
	17	277	459	0.319	3.19	0.733
	18	302	427	0.349	2.93	0.742
	19	296	425	0.344	2.98	0.745
	20	297	436	0.343	2.99	0.748
	21	289	425	0.337	3.08	0.730
	22	312	449	0.354	2.95	0.742
	23	283	454	0.326	3.04	0.746
	24	293	452	0.336	3.13	0.750
	25	293	452	0.336	3.04	0.746
	26	326	417	0.374	2.74	0.767
	27	290	459	0.332	3.11	0.741
	28	290	449	0.334	3.24	0.754
	29	285	446	0.329	3.06	0.749
	30	235	254	0.316	3.15	0.748
3	1	412	432	0.447	1.64	0.822
	2	379	327	0.444	1.56	0.813
	3	367	353	0.427	1.78	0.812
	4	376	372	0.430	1.66	0.826
	5	424	475	0.447	1.71	0.827
	6	344	311	0.416	1.69	0.817
	7	435	440	0.464	1.68	0.823
	8	444	455	0.468	1.62	0.825
	9	401	416	0.441	1.79	0.808
	10	404	402	0.447	1.66	0.824
	11	427	430	0.460	1.72	0.826
	12	361	427	0.404	1.78	0.804
	13	433	452	0.459	1.66	0.832
	14	407	434	0.442	1.72	0.829
	15	433	418	0.468	1.65	0.828
	16	453	481	0.468	1.75	0.828
	17	439	404	0.476	1.64	0.817

	18	404	450	0.436	1.72	0.818
	19	418	438	0.450	1.68	0.826
	20	429	487	0.448	1.72	0.823
	21	398	408	0.441	1.66	0.817
	22	412	455	0.441	1.80	0.808
	23	398	404	0.442	1.68	0.826
	24	427	463	0.452	1.71	0.832
	25	368	365	0.425	1.71	0.817
	26	426	406	0.465	1.60	0.823
	27	388	389	0.437	1.72	0.818
	28	431	393	0.473	1.63	0.833
	29	407	440	0.441	1.71	0.825
	30	399	386	0.447	1.69	0.823
	1	536	459	0.537	1.48	0.871
	2	549	446	0.550	1.45	0.873
	3	542	449	0.544	1.49	0.875
	4	529	455	0.533	1.48	0.875
	5	542	421	0.552	1.44	0.874
	6	522	401	0.543	1.41	0.877
	7	529	446	0.536	1.46	0.876
	8	513	448	0.523	1.45	0.863
	9	532	410	0.548	1.44	0.869
	10	543	455	0.544	1.43	0.880
	11	556	437	0.558	1.50	0.882
	12	536	434	0.544	1.48	0.871
	13	527	438	0.536	1.48	0.865
	14	538	387	0.559	1.42	0.876
4	15	503	378	0.535	1.42	0.875
	16	546	387	0.565	1.43	0.882
	17	540	414	0.553	1.40	0.876
	18	533	418	0.546	1.45	0.885
	19	531	459	0.534	1.47	0.876
	20	502	355	0.541	1.43	0.870
	21	561	468	0.553	1.48	0.880
	22	514	384	0.542	1.44	0.875
	23	530	377	0.556	1.39	0.871
	24	544	407	0.558	1.42	0.875
	25	504	451	0.516	1.53	0.861
	26	549	450	0.549	1.49	0.881
	27	532	463	0.533	1.54	0.877
	28	525	426	0.538	1.47	0.870
	29	502	381	0.533	1.46	0.873
	30	522	456	0.528	1.46	0.870
	1	633	463	0.604	1.36	0.906
	2	652	464	0.616	1.33	0.910
	3	658	473	0.618	1.36	0.908
5	4	594	389	0.599	1.31	0.907
	5	641	476	0.606	1.39	0.907
	6	635	415	0.620	1.32	0.912
	7	634	382	0.629	1.30	0.909

	8	653	463	0.617	1.34	0.913
	9	604	352	0.618	1.27	0.911
	10	631	432	0.612	1.34	0.908
	11	639	374	0.635	1.31	0.914
	12	635	419	0.618	1.35	0.911
	13	658	460	0.621	1.33	0.912
	14	638	405	0.625	1.29	0.913
	15	654	415	0.632	1.32	0.916
	16	641	434	0.618	1.33	0.906
	17	629	429	0.611	1.32	0.909
	18	605	352	0.618	1.29	0.909
	19	654	449	0.622	1.35	0.917
	20	651	430	0.626	1.36	0.912
	21	639	463	0.608	1.38	0.917
	22	624	465	0.597	1.36	0.901
	23	604	403	0.602	1.34	0.899
	24	642	507	0.597	1.39	0.904
	25	625	397	0.618	1.31	0.900
	26	655	435	0.627	1.32	0.916
	27	641	424	0.621	1.34	0.911
	28	616	461	0.593	1.33	0.898
	29	629	426	0.612	1.33	0.910
	30	620	376	0.621	1.30	0.905

Table S1.6 Simulation results of DEF-scRNA-seq under different biological replicates (K).

	DEF-scRNA-seq					
K	Round	TP	FP	F1-score	MRSR	AUC
	1	384	616	0.384	2.10	0.767
	2	378	622	0.378	2.20	0.773
	3	371	629	0.371	2.26	0.757
	4	369	631	0.369	2.26	0.768
	5	373	627	0.373	2.19	0.771
	6	373	627	0.373	2.35	0.757
	7	380	620	0.380	2.15	0.781
	8	404	596	0.404	2.10	0.782
	9	374	626	0.374	2.23	0.773
	10	404	596	0.404	2.12	0.780
2	11	407	593	0.407	2.07	0.778
	12	379	621	0.379	2.16	0.774
	13	380	620	0.380	2.22	0.781
	14	383	617	0.383	2.14	0.762
	15	385	615	0.385	2.20	0.770
	16	357	643	0.357	2.35	0.761
	17	380	620	0.380	2.20	0.759
	18	383	617	0.383	2.11	0.769
	19	376	624	0.376	2.15	0.769
	20	372	628	0.372	2.08	0.770
	21	377	623	0.377	2.16	0.762

	22	399	601	0.399	2.08	0.768
	23	370	630	0.370	2.28	0.768
	24	374	626	0.374	2.15	0.776
	25	369	631	0.369	2.11	0.771
	26	406	594	0.406	2.01	0.777
	27	370	630	0.370	2.19	0.758
	28	388	612	0.388	2.20	0.779
	29	396	604	0.396	2.10	0.778
	30	379	621	0.379	2.11	0.777
3	1	507	493	0.507	1.59	0.851
	2	495	505	0.495	1.55	0.847
	3	487	513	0.487	1.66	0.838
	4	490	510	0.490	1.59	0.850
	5	507	493	0.507	1.60	0.852
	6	469	531	0.469	1.75	0.837
	7	506	494	0.506	1.59	0.853
	8	507	493	0.507	1.56	0.847
	9	481	519	0.481	1.72	0.834
	10	497	503	0.497	1.64	0.851
	11	493	507	0.493	1.60	0.853
	12	464	536	0.464	1.72	0.837
	13	510	490	0.510	1.57	0.853
	14	478	522	0.478	1.71	0.848
	15	493	507	0.493	1.62	0.850
	16	509	491	0.509	1.61	0.855
	17	494	506	0.494	1.64	0.840
	18	503	497	0.503	1.58	0.841
	19	504	496	0.504	1.55	0.847
	20	514	486	0.514	1.62	0.852
	21	496	504	0.496	1.60	0.839
	22	493	507	0.493	1.66	0.838
	23	491	509	0.491	1.61	0.846
	24	513	487	0.513	1.60	0.859
	25	486	514	0.486	1.64	0.839
	26	508	492	0.508	1.59	0.843
	27	490	510	0.490	1.63	0.845
	28	509	491	0.509	1.58	0.856
	29	512	488	0.512	1.64	0.855
	30	507	493	0.507	1.56	0.849
4	1	593	407	0.593	1.33	0.900
	2	596	404	0.596	1.34	0.905
	3	585	415	0.585	1.37	0.892
	4	596	404	0.596	1.39	0.894
	5	606	394	0.606	1.32	0.902
	6	613	387	0.613	1.35	0.901
	7	608	392	0.608	1.33	0.901
	8	585	415	0.585	1.39	0.885
	9	608	392	0.608	1.37	0.892
	10	604	396	0.604	1.31	0.903
	11	602	398	0.602	1.38	0.902

	12	602	398	0.602	1.33	0.898
	13	580	420	0.580	1.40	0.885
	14	591	409	0.591	1.38	0.894
	15	601	399	0.601	1.38	0.900
	16	598	402	0.598	1.38	0.904
	17	602	398	0.602	1.34	0.899
	18	598	402	0.598	1.36	0.907
	19	591	409	0.591	1.34	0.897
	20	584	416	0.584	1.37	0.895
	21	619	381	0.619	1.34	0.906
	22	612	388	0.612	1.36	0.896
	23	592	408	0.592	1.33	0.892
	24	618	382	0.618	1.33	0.898
	25	579	421	0.579	1.40	0.884
	26	592	408	0.592	1.38	0.900
	27	596	404	0.596	1.39	0.904
	28	602	398	0.602	1.37	0.890
	29	586	414	0.586	1.36	0.897
	30	591	409	0.591	1.39	0.896
5	1	661	339	0.661	1.25	0.930
	2	666	334	0.666	1.22	0.933
	3	668	332	0.668	1.24	0.923
	4	665	335	0.665	1.23	0.931
	5	658	342	0.658	1.27	0.926
	6	666	334	0.666	1.23	0.932
	7	675	325	0.675	1.23	0.930
	8	674	326	0.674	1.20	0.934
	9	660	340	0.660	1.20	0.931
	10	672	328	0.672	1.23	0.930
	11	674	326	0.674	1.22	0.933
	12	660	340	0.660	1.25	0.930
	13	658	342	0.658	1.21	0.936
	14	669	331	0.669	1.23	0.932
	15	672	328	0.672	1.20	0.937
	16	648	352	0.648	1.24	0.926
	17	670	330	0.670	1.24	0.928
	18	653	347	0.653	1.23	0.927
	19	655	345	0.655	1.24	0.934
	20	672	328	0.672	1.22	0.929
	21	658	342	0.658	1.25	0.937
	22	659	341	0.659	1.23	0.925
	23	660	340	0.660	1.24	0.919
	24	661	339	0.661	1.23	0.928
	25	651	349	0.651	1.25	0.922
	26	683	317	0.683	1.22	0.932
	27	679	321	0.679	1.21	0.931
	28	659	341	0.659	1.26	0.927
	29	667	333	0.667	1.23	0.932
	30	661	339	0.661	1.21	0.928

Table S1.7 Simulation results of DEsingle under different biological replicates (K).

			DEsingle			
K	Round	TP	FP	F1-score	MRSR	AUC
2	1	176	196	0.257	1.83	0.727
	2	161	189	0.239	1.79	0.739
	3	152	192	0.226	1.93	0.722
	4	171	182	0.253	1.85	0.733
	5	186	199	0.269	1.79	0.746
	6	180	174	0.266	1.80	0.730
	7	203	179	0.294	1.72	0.752
	8	205	198	0.292	1.65	0.739
	9	173	203	0.251	1.86	0.730
	10	196	200	0.281	1.78	0.733
	11	190	160	0.281	1.71	0.729
	12	169	167	0.253	1.72	0.736
	13	187	209	0.268	1.86	0.746
	14	187	181	0.273	1.83	0.734
	15	184	193	0.267	1.78	0.735
	16	172	186	0.253	1.78	0.727
	17	174	209	0.252	1.84	0.724
	18	197	177	0.287	1.76	0.731
	19	161	181	0.240	1.78	0.733
	20	173	176	0.256	1.67	0.739
	21	159	184	0.237	1.78	0.724
	22	188	197	0.271	1.79	0.734
	23	174	171	0.259	1.73	0.739
	24	181	182	0.266	1.74	0.738
	25	186	175	0.273	1.71	0.736
	26	221	179	0.316	1.63	0.744
	27	175	192	0.256	1.69	0.725
	28	172	187	0.253	1.80	0.743
	29	184	205	0.265	1.84	0.728
	30	209	168	0.304	1.59	0.735
3	1	285	212	0.381	1.41	0.801
	2	285	198	0.384	1.46	0.795
	3	264	199	0.361	1.57	0.788
	4	274	237	0.363	1.51	0.805
	5	285	237	0.375	1.55	0.808
	6	277	212	0.372	1.55	0.787
	7	298	201	0.398	1.48	0.799
	8	292	203	0.391	1.42	0.802
	9	251	200	0.346	1.60	0.789
	10	296	189	0.399	1.47	0.805
	11	287	207	0.384	1.54	0.803
	12	275	235	0.364	1.57	0.780
	13	325	233	0.417	1.46	0.803
	14	281	214	0.376	1.51	0.808
	15	298	193	0.400	1.44	0.802
	16	295	211	0.392	1.47	0.812
	17	304	208	0.402	1.48	0.801

	18	286	230	0.377	1.54	0.796
	19	295	192	0.397	1.39	0.795
	20	300	236	0.391	1.46	0.804
	21	282	217	0.376	1.46	0.794
	22	279	211	0.374	1.57	0.793
	23	262	194	0.360	1.44	0.800
	24	299	231	0.391	1.51	0.809
	25	273	217	0.366	1.54	0.798
	26	306	207	0.404	1.42	0.804
	27	277	203	0.374	1.54	0.795
	28	305	197	0.406	1.40	0.804
	29	285	218	0.379	1.49	0.801
	30	289	207	0.386	1.46	0.803
4	1	366	239	0.456	1.38	0.846
	2	410	241	0.497	1.39	0.850
	3	383	235	0.473	1.40	0.842
	4	398	232	0.488	1.36	0.851
	5	388	237	0.478	1.36	0.846
	6	391	218	0.486	1.30	0.853
	7	389	222	0.483	1.32	0.847
	8	399	245	0.485	1.36	0.833
	9	385	250	0.471	1.33	0.843
	10	400	225	0.492	1.30	0.853
	11	381	263	0.464	1.41	0.851
	12	388	232	0.479	1.37	0.840
	13	367	249	0.454	1.41	0.841
	14	400	223	0.493	1.37	0.849
	15	373	224	0.467	1.35	0.845
	16	393	230	0.484	1.40	0.850
	17	416	219	0.509	1.29	0.844
	18	383	220	0.478	1.33	0.855
	19	385	237	0.475	1.36	0.846
	20	378	237	0.468	1.34	0.841
	21	399	239	0.487	1.38	0.854
	22	367	230	0.460	1.36	0.845
	23	389	221	0.483	1.31	0.842
	24	384	212	0.481	1.35	0.852
	25	354	248	0.442	1.44	0.836
	26	380	243	0.468	1.41	0.851
	27	395	248	0.481	1.43	0.846
	28	378	228	0.471	1.34	0.840
	29	358	229	0.451	1.38	0.839
	30	392	253	0.477	1.38	0.846
5	1	474	257	0.548	1.31	0.884
	2	483	259	0.555	1.27	0.884
	3	468	291	0.532	1.34	0.877
	4	465	284	0.532	1.28	0.883
	5	451	276	0.522	1.36	0.876
	6	475	256	0.549	1.29	0.885
	7	475	244	0.553	1.28	0.885

	8	452	253	0.530	1.25	0.883
	9	464	247	0.542	1.31	0.881
	10	453	227	0.539	1.29	0.880
	11	470	261	0.543	1.32	0.889
	12	476	259	0.549	1.31	0.882
	13	477	244	0.554	1.28	0.888
	14	488	243	0.564	1.25	0.888
	15	485	242	0.562	1.27	0.883
	16	477	249	0.553	1.28	0.875
	17	461	252	0.538	1.27	0.886
	18	456	245	0.536	1.27	0.884
	19	467	282	0.534	1.32	0.886
	20	457	269	0.530	1.34	0.877
	21	466	263	0.539	1.32	0.890
	22	461	282	0.529	1.33	0.876
	23	439	256	0.518	1.31	0.870
	24	483	247	0.558	1.33	0.879
	25	456	264	0.530	1.31	0.875
	26	491	260	0.561	1.26	0.889
	27	469	272	0.539	1.29	0.882
	28	489	251	0.562	1.25	0.877
	29	467	276	0.536	1.28	0.880
	30	465	246	0.544	1.26	0.879

Table S1.8 Simulation results of ROSeq under different biological replicates (K).

ROSeq						
K	Round	TP	FP	F1-score	MRSR	AUC
2	1	271	1348	0.207	5.24	0.469
	2	266	1419	0.198	5.48	0.448
	3	271	1347	0.207	5.07	0.476
	4	241	1398	0.183	6.07	0.451
	5	292	1366	0.220	4.98	0.474
	6	263	1375	0.199	5.19	0.471
	7	282	1378	0.212	5.30	0.468
	8	247	1358	0.190	5.53	0.467
	9	269	1405	0.201	5.89	0.462
	10	277	1370	0.209	5.06	0.477
	11	264	1359	0.201	5.45	0.468
	12	267	1349	0.204	5.37	0.473
	13	281	1337	0.215	5.04	0.477
	14	243	1378	0.185	5.71	0.450
	15	280	1395	0.209	5.52	0.473
	16	252	1341	0.194	5.93	0.458
	17	284	1423	0.210	5.38	0.469
	18	238	1411	0.180	6.00	0.468
	19	255	1378	0.194	5.88	0.459
	20	253	1370	0.193	5.78	0.455
	21	276	1371	0.209	5.29	0.454
	22	274	1384	0.206	5.50	0.471

	23	245	1329	0.190	5.46	0.466
	24	257	1320	0.199	5.34	0.463
	25	242	1349	0.187	5.89	0.462
	26	251	1387	0.190	5.71	0.464
	27	261	1366	0.199	5.27	0.450
	28	269	1405	0.201	5.33	0.473
	29	271	1343	0.207	5.24	0.465
	30	255	1358	0.195	5.72	0.460
3	1	283	969	0.251	3.87	0.632
	2	293	998	0.256	3.50	0.609
	3	316	969	0.277	3.50	0.633
	4	288	944	0.258	3.54	0.613
	5	312	975	0.273	3.33	0.625
	6	289	993	0.253	3.76	0.621
	7	311	947	0.275	3.40	0.631
	8	268	987	0.238	3.90	0.621
	9	269	970	0.240	3.71	0.608
	10	299	960	0.265	3.62	0.620
	11	335	1008	0.286	3.38	0.610
	12	312	1021	0.267	3.57	0.620
	13	318	994	0.275	3.57	0.612
	14	262	962	0.236	4.19	0.619
	15	331	948	0.290	3.17	0.635
	16	303	991	0.264	3.91	0.614
	17	296	1016	0.256	3.81	0.628
	18	282	985	0.249	3.67	0.609
	19	325	1042	0.275	3.56	0.607
	20	298	1029	0.256	3.81	0.607
	21	306	995	0.266	3.62	0.614
	22	312	976	0.273	3.32	0.612
	23	317	1027	0.270	3.30	0.601
	24	303	1021	0.261	3.61	0.624
	25	286	930	0.258	3.51	0.626
	26	281	985	0.248	3.84	0.645
	27	308	1005	0.266	3.49	0.621
	28	303	965	0.267	3.55	0.629
	29	308	1000	0.267	3.64	0.616
	30	284	1040	0.244	3.87	0.616
4	1	326	764	0.312	2.66	0.726
	2	368	750	0.347	2.54	0.726
	3	337	799	0.316	2.64	0.729
	4	361	732	0.345	2.54	0.722
	5	356	765	0.336	2.55	0.710
	6	335	751	0.321	2.71	0.724
	7	356	730	0.341	2.44	0.718
	8	340	768	0.323	2.59	0.725
	9	326	756	0.313	2.65	0.703
	10	329	775	0.313	2.72	0.730
	11	353	740	0.337	2.50	0.710
	12	349	721	0.337	2.48	0.717

	13	320	771	0.306	2.78	0.727
	14	336	690	0.332	2.52	0.702
	15	364	715	0.350	2.53	0.726
	16	360	764	0.339	2.45	0.718
	17	311	766	0.299	2.66	0.712
	18	375	760	0.351	2.47	0.709
	19	322	691	0.320	2.50	0.711
	20	349	720	0.337	2.59	0.712
	21	343	749	0.328	2.57	0.718
	22	328	746	0.316	2.64	0.726
	23	347	721	0.336	2.61	0.704
	24	330	769	0.314	2.65	0.720
	25	347	707	0.338	2.45	0.725
	26	359	688	0.351	2.48	0.720
	27	347	749	0.331	2.59	0.723
	28	350	753	0.333	2.51	0.712
	29	340	788	0.320	2.79	0.717
	30	314	719	0.309	2.69	0.697
5	1	419	511	0.434	1.80	0.788
	2	443	501	0.456	1.73	0.805
	3	430	498	0.446	1.70	0.806
	4	454	485	0.468	1.62	0.812
	5	448	461	0.469	1.64	0.812
	6	429	482	0.449	1.80	0.807
	7	432	492	0.449	1.77	0.804
	8	414	488	0.435	1.70	0.805
	9	432	481	0.452	1.70	0.797
	10	431	472	0.453	1.68	0.805
	11	448	492	0.462	1.71	0.809
	12	414	477	0.438	1.74	0.803
	13	432	497	0.448	1.76	0.801
	14	435	482	0.454	1.66	0.801
	15	439	510	0.450	1.60	0.807
	16	414	507	0.431	1.71	0.800
	17	434	495	0.450	1.77	0.804
	18	436	497	0.451	1.73	0.814
	19	445	483	0.462	1.74	0.798
	20	408	493	0.429	1.77	0.785
	21	414	474	0.439	1.74	0.808
	22	436	473	0.457	1.63	0.803
	23	444	468	0.464	1.63	0.817
	24	423	494	0.441	1.77	0.801
	25	429	485	0.448	1.67	0.807
	26	436	496	0.451	1.68	0.804
	27	442	503	0.454	1.71	0.803
	28	447	473	0.466	1.61	0.816
	29	411	487	0.433	1.75	0.800
	30	435	458	0.460	1.65	0.805
